# Iridium single atoms incorporated in Co_3_O_4_ efficiently catalyze the oxygen evolution in acidic conditions

**DOI:** 10.1038/s41467-022-35426-8

**Published:** 2022-12-14

**Authors:** Yiming Zhu, Jiaao Wang, Toshinari Koketsu, Matthias Kroschel, Jin-Ming Chen, Su-Yang Hsu, Graeme Henkelman, Zhiwei Hu, Peter Strasser, Jiwei Ma

**Affiliations:** 1 Shanghai Key Laboratory for R&D and Application of Metallic Functional Materials, Institute of New Energy for Vehicles, School of Materials Science and Engineering, Tongji University, Shanghai, 201804 China; 2 Department of Chemistry and the Oden Institute for Computational Engineering and Sciences, The University of Texas at Austin, Austin, TX 78712-0165 USA; 3 Department of Chemistry, Technical University Berlin, 10623 Berlin, Germany; 4 National Synchrotron Radiation Research Center, Hsinchu, 30076 Taiwan; 5 Max Planck Institute for Chemical Physics of Solids, Nöthnitzer Strasse 40, 01187 Dresden, Germany

**Keywords:** Nanoscale materials, Electrocatalysis, Electrocatalysis

## Abstract

Designing active and stable electrocatalysts with economic efficiency for acidic oxygen evolution reaction is essential for developing proton exchange membrane water electrolyzers. Herein, we report on a cobalt oxide incorporated with iridium single atoms (Ir-Co_3_O_4_), prepared by a mechanochemical approach. Operando X-ray absorption spectroscopy reveals that Ir atoms are partially oxidized to active Ir^>4+^ during the reaction, meanwhile Ir and Co atoms with their bridged electrophilic O ligands acting as active sites, are jointly responsible for the enhanced performance. Theoretical calculations further disclose the isolated Ir atoms can effectively boost the electronic conductivity and optimize the energy barrier. As a result, Ir-Co_3_O_4_ exhibits significantly higher mass activity and turnover frequency than those of benchmark IrO_2_ in acidic conditions. Moreover, the catalyst preparation can be easily scaled up to gram-level per batch. The present approach highlights the concept of constructing single noble metal atoms incorporated cost-effective metal oxides catalysts for practical applications.

## Abstract (web summary)

Designing active, stable and cost-effective catalysts for the acidic oxygen evolution reaction remains a challenge. Here, the authors report iridium single atoms incorporated cobalt oxides, showing distinctly enhanced performance in the acid.

## Introduction

Centuries of industrialization and human activities have consumed enormous amounts of fossil fuels, which has inevitably led to the current energy crisis and global warming^1^. Hydrogen (H_2_) is considered a promising alternative energy carrier of the future owing to its high energy density and potential for carbon-free emission^2^. Among many H_2_ production methods, water electrolysis has received much attention as a feasible technology for practical applications. Nevertheless, due to the sluggish kinetics of the complex four-electron transfer process, the oxygen evolution reaction (OER) at the anode side of the water electrolyzer demonstrates high overpotentials, which severely limit the overall operation efficiency and impede the large commercialization of water electrolyzers^3–6^. Therefore, tremendous effort has been devoted to the rational design of robust electrocatalysts which improve the OER activity and energy conversion efficiency.

Over the past few decades, the noble metal oxides based on iridium (Ir) and ruthenium (Ru) are considered the state-of-the-art OER electrocatalysts. However, their widespread applications are limited by the cost and scarcity of Ir and Ru^7–10^. As a result, the first row 3*d* transition metal oxides and their derivatives, with merits of high abundance and low cost have received much attention as alternative OER candidates^11–15^. Among all 3*d* transition metal oxides, cobalt-based oxides such as spinel Co_3_O_4_ have been widely used to activate OER activity, as demonstrated by both experimental data and theoretical calculations^16–19^. Nevertheless, Co_3_O_4_ still suffers from an overpotential of more than 400 mV for the OER at a current density of 10 mA cm^−2^, which is far from meeting the requirements for practical applications. Moreover, Co_3_O_4_ is unstable under the harsh OER conditions at the high oxidizing potential in corrosive media such as an acidic electrolyte^20–22^. It is well known that the 4*d*/5*d* noble elements possess a large *d*-electronic wave-function spatial extent, generating versatile electronic structures via the interaction between 3*d* and 4*d*/5*d* orbitals, which is beneficial for enhancing OER activity. One available method that has been frequently used is incorporating bulk amounts of noble metals with Co or its derivatives to obtain hybrid 3*d*/4*d* or 3*d*/5*d* nanomaterials as OER electrocatalysts^23–25^. In this regard, Li et al. reported that the Sr_2_CoIrO_6-δ_, which benefits from a synergy between Co and Ir active sites, exhibits a low overpotential toward OER^26^. Pi and co-workers also demonstrated that IrCo bimetallic nanoclusters can be employed as efficient OER electrocatalysts^27^. In addition, Shah et al. developed a catalyst with single Co atoms doped on RuO_2_ sphere, yielding remarkable catalytic performances for water splitting^28^. Notwithstanding these efforts to modify Co-based nanomaterials as alternative OER catalysts, it remains difficult to tip the balance between low cost and high performance. Recently, the strategy of constructing highly dispersed single-sites catalysts has attracted significantly attentions for a range of electrocatalytic reactions^29^. Downsizing the materials into isolated atoms is beneficial for reducing the amount of bulk metal, which can greatly maximize the atom-utilization efficiency, leading to remarkable catalytic mass activity^30–32^. Additionally, the unique electronic structure of uniformly dispersed active sites usually results in a strong interaction with the host material, effectively adjusting the electrochemical microenvironment over catalysts and potentially boosting the electrocatalytic activity^33–35^. Motivated by the abovementioned guidelines, the integration of spinel Co-based oxide with noble metal single atoms as the prospective OER electrocatalyst, possesses advantages of minimized noble metal usage and optimized catalytic performances. Nevertheless, the large lattice discrepancy between the diverse atoms and strong binding energy between the same atoms, are great challenges to disperse homogeneous noble metal single sites in the transition metal oxide host^36^. And the production of traditional methods to prepare the single-atom materials such as impregnation are usually limited to milligram level, it is extremely urgent to explore a mass-production method to meet the requirements for practical applications.

In this work, we deploy a facile and economical strategy to prepare atomically dispersed Ir atoms doped in spinel Co_3_O_4_ (Ir-Co_3_O_4_) via a mechanochemical method. By introducing a trace amount of Ir (~1.05 at%), Ir-Co_3_O_4_ displays a remarkable OER overpotential of 236 mV in an acid medium at the current density of 10 mA cm^−2^, which is significantly lower than that of as-prepared Co_3_O_4_ (412 mV). Significantly, both the normalized mass activity and TOF of Ir-Co_3_O_4_ can reach almost two order magnitudes higher than those of commercial IrO_2_ at an overpotential of 300 mV, respectively. Meanwhile, the stability can also be extended much longer after incorporating Ir single atoms within the lattice of Co_3_O_4_. Using operando X-ray absorption near edge structure (XANES) at the Ir-*L*_3_ and Co-*K* edges, it is observed that the Ir species are partially oxidized to OER-active Ir^>4+^, and the elevated valence states with increased voltage also indicate that both high-valence Ir and Co atoms with their bridged electrophilic O ligands serve as active sites, which are jointly responsible for charge transfer in electrochemical reactions. In addition, density functional theory (DFT) calculations confirm the improved electronic conductivity and facile energy barrier contributed by the incorporated Ir single atoms, resulting in a high OER catalytic activity. Moreover, the preparation of Ir-Co_3_O_4_ can be easily scaled up to achieve gram-level per batch with negligible activity loss, demonstrating this strategy as an economical approach to prepare advanced electrocatalysts in a larger scale for practical applications.

## Results

### Structural characterizations of Ir-Co_3_O_4_

**Fig. 1:**
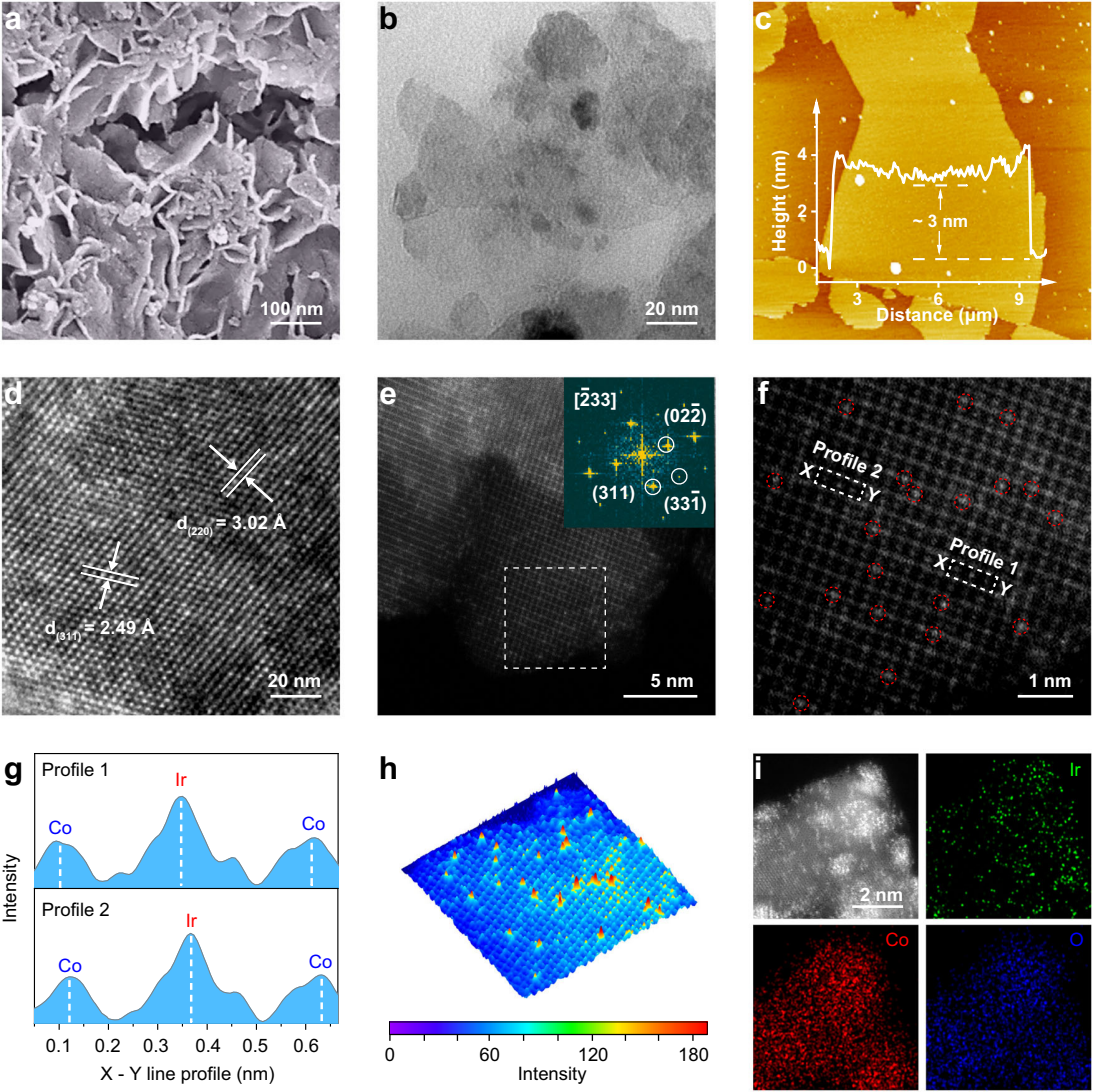
Structural characterizations of Ir-Co_3_O_4_. **a** SEM, **(b**) TEM, **(c)** AFM and (**d**) HR-TEM images of Ir-Co_3_O_4_. Inset in (**c**) is the height profile of Ir-Co_3_O_4_ along the dashed line. **e** AC HAADF-STEM image and the corresponding FFT image (inset) of Ir-Co_3_O_4_. **f** The enlarged area in (**e**), with the Ir single atoms marked in circles, and (**g**) intensity profiles along the dashed rectangles. **h** 3D atom-overlapping Gaussian function fitting mapping of the selected area in (**e**). **i** AC HAADF-STEM image and the corresponding elemental mappings of Ir-Co_3_O_4_.

Ir-Co_3_O_4_ was synthesized based on the mechanochemical method, in which a solid solution of NaCl and metal precursors were formed by ball milling and then calcination (see Methods for details). The morphology of Ir-Co_3_O_4_ was initially characterized by scanning electron microscopy (SEM). As shown in Fig. 1a, Ir-Co_3_O_4_ reveals nanosized and flake morphology. Typical transmission electron microscopy (TEM) images show that Ir-Co_3_O_4_ is structured as flexible ultrathin nanosheets with thickness of about 3 nm as indicated by atomic force microscopy (AFM) images. The ultrathin 2D structure of Ir-Co_3_O_4_ can potentially maximize the exposed active sites on the surface and increase the contact area with the electrolyte, leading to the enhanced catalytic performance (Fig. 1b, c & Supplementary Fig. 1)^37^. Detailed structural analysis by high-resolution TEM (HR-TEM) confirms that Ir-Co_3_O_4_ has lattice spacings of 2.48 Å and 3.02 Å, which can be ascribed to the Co_3_O_4_ (311) and (220) facets, respectively. Furthermore, due to the introduction of NaCl as the hard template in preparation, pores are generated and can be observed in the lattice structure of Ir-Co_3_O_4_. In general, those mesopores often result in more exposed active atoms on the edge, which are favorable for catalytic activity (Fig. 1d & Supplementary Fig. 2)^38^. Meanwhile, pure Co_3_O_4_ synthesized by the same method exhibits no evident differences in morphology as compared with Ir-Co_3_O_4_ (Supplementary Fig. 3). The crystal structures of Ir-Co_3_O_4_ and as-synthesized Co_3_O_4_ were characterized by X-ray diffraction (XRD) analysis, revealing similar patterns of face-centered cubic symmetry (space group: *Fd-3 m*) of the spinel structure. Rietveld refinements were further performed on those XRD patterns: the refined lattice parameters are *a* = 8.0972(5) Å, *b* = 8.0972(5) Å, *c* = 8.0972(5) Å, and *V* = 530.89(6) Å^3^ for Ir-Co_3_O_4_, as compared to *a* = 8.0754(3) Å, *b* = 8.0754(3) Å, *c* = 8.0754(3) Å, and *V* = 526.61(3) Å^3^ for as-prepared Co_3_O_4_ (Supplementary Figs. 4, 5 & Supplementary Table 1, 2). The expanded crystal cells of Ir-Co_3_O_4_ may be attributed to Ir atoms with a larger radius incorporated into the lattice of Co_3_O_4_. Subsequent composition evaluation of Ir-Co_3_O_4_ by inductively coupled plasma-optical emission spectrometry (ICP-OES) and SEM energy-dispersive spectroscopy (SEM-EDS) confirms that Ir has an atomic ratio of 1.05% (Supplementary Fig. 6). In addition, the possible Fe impurity in Ir-Co_3_O_4_ derived from the stainless-steel ball milling device was also determined to be 0.0667 wt% by ICP-OES, which can be ignored compared to the contents of other elements.

To determine the distribution of Ir species at the atomic scale, aberration corrected high angle annular dark field-scanning transmission electron microscopy (AC HAADF-STEM) images were collected on Ir-Co_3_O_4_. The corresponding fast Fourier transform (FFT) image identifies the exposure of (33$\mathop{1}\limits^{-}$), (311) and (02$\mathop{2}\limits^{-}$) planes of Co_3_O_4_ along the [$\mathop{2}\limits^{-}$33] zone axis. Interestingly, high-magnification images demonstrate the presence of monodispersed Ir single atoms (bright dots marked by red circles in Fig. 1e, f). On account of the higher Z number, the Ir atom brightness is more intense than neighboring Co atoms^39^. The dispersion of isolated Ir atoms was further verified by the profiles along the dashed rectangles in the AC HAADF-STEM image, in which the different atomic intensities can be clearly distinguished (Fig. 1g). Additionally, the 3D atom-overlapping Gaussian function fitting mapping of the selected area in Fig. 1e is employed to observe the atomic distribution. As shown in Fig. 1h, the highest peaks representing Ir atoms are evenly dispersed, providing further evidence of isolated atoms. Elemental mapping images also show a homogeneous dispersion of Ir, Co, and O atoms throughout the entire Ir-Co_3_O_4_ sample (Fig. 1i & Supplementary Fig. 7).

### Electronic and atomic structure analysis of Ir-Co_3_O_4_

**Fig. 2:**
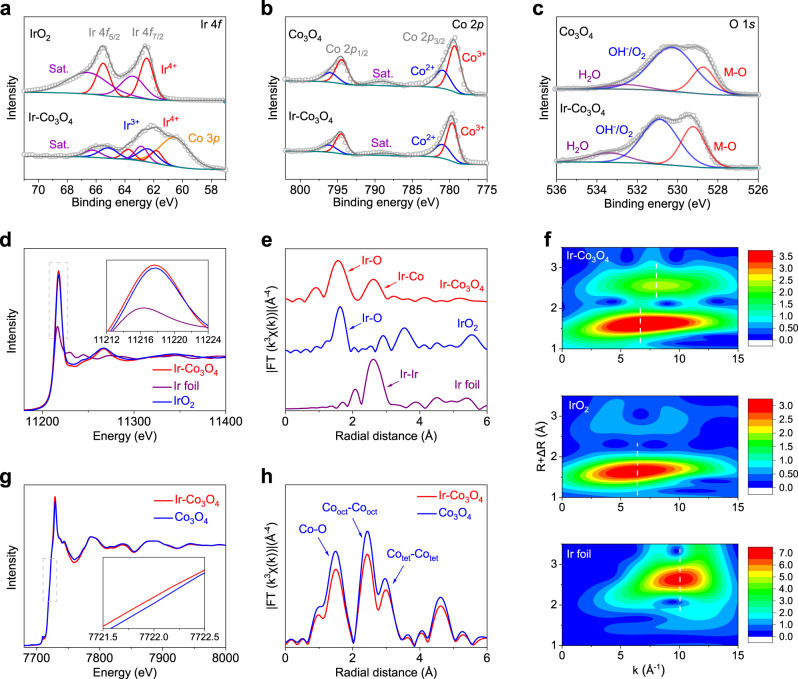
Electronic and atomic structure analysis of Ir-Co_3_O_4_. **a** Ir 4*f* XPS spectra of Ir-Co_3_O_4_ and IrO_2_. **b** Co 2*p* XPS spectra and (**c**) O 1 *s* XPS spectra of Ir-Co_3_O_4_ and Co_3_O_4_. **d** XANES spectra of Ir-*L*_3_ edge on Ir-Co_3_O_4_, Ir foil and IrO_2_. **d** The normalized Ir-*L*_3_ edge XANES spectra of Ir-Co_3_O_4_, Ir foil and IrO_2_. **e** Ir-*L*_3_ edge EXAFS spectra and **(f**) corresponding wavelet-transformed *k*^3^-weighted EXAFS spectra of Ir-Co_3_O_4_, Ir foil and IrO_2_. **g** The normalized Co-*K* edge XANES spectra and (**h**) Co-*K* edge XANES spectra of Ir-Co_3_O_4_ and Co_3_O_4_.

To understand the electronic structure of Ir-Co_3_O_4_, we carried out X-ray photoelectron spectroscopy (XPS). All XPS spectra were calibrated with C 1*s* using a binding energy of 284.8 eV. The survey XPS spectra of Ir-Co_3_O_4_ demonstrate the existence of Ir, Co and O elements on the surface (Supplementary Fig. 8). As shown in Fig. 2a, a typical IrO_2_ XPS shows one sets of doublets at 62.5 and 65.4 eV, which can be attributed to Ir^4+^. For Ir-Co_3_O_4_, the Ir 4*f* spectrum can be deconvoluted into two sets of doublets centered at 61.8 / 63.7 eV and 62.4 / 65.3 eV, which can be attributed to Ir^4+^ and Ir^3+^, respectively^40–42^. Accordingly, the Ir species on the surface of Ir-Co_3_O_4_ is oxidized with chemical states between +3 and +4. The peak located at 60.6 eV is produced by Co 3*p*. The introduction of Ir affects the electronic structure of Co_3_O_4_; as revealed in Fig. 2b, the main peaks of Co 2*p*_3/2_ of both Ir-Co_3_O_4_ and as-synthesized Co_3_O_4_ present signals that can be identified as Co^2+^ and Co^3+^, respectively. Nevertheless, the Co^2+^ over Co^3+^ ratio is increased by 8.33 % after Ir species are introduced (the ratios of Co^2+^: Co^3+^ in Co_3_O_4_ and Ir-Co_3_O_4_ are 0.48: 1 and 0.52: 1, respectively), suggesting a decreased valence state of Co on the surface of Ir-Co_3_O_4_. Moreover, since the incorporated Ir possesses stronger covalency, the O 1*s* XPS spectrum of Ir-Co_3_O_4_ reveals a more positive binding energy and larger lattice oxygen M-O concentration than that of Co_3_O_4_, which is induced by the incorporation of Ir atoms (Fig. 2c)^43^.

X-ray absorption spectroscopy (XAS) was conducted with the aim of distinguishing the electronic structure and the local atomic environment of Ir-Co_3_O_4_. The energy position of the white line of X-ray absorption near edge structure (XANES) at the 5*d* transition metals is highly sensitive to their valence states^44–48^. And it is well known that an increase of the valence state of the 5*d* metal ion by one results in a shift of the *L*_2,3_ XAS spectra by one or more eV toward higher energies^44^. Figure 2d shows the Ir *L*_3_ XANES of Ir-Co_3_O_4_ together with Ir foil and IrO_2_ for comparison. One can observe that the white line of Ir-Co_3_O_4_ shifts by 1.6 eV to a higher energy relative to Ir foil, but is located extremely close to that of IrO_2_, demonstrating that the Ir chemical state is close to +4, which is distinct from those in nanoparticles or supported single atoms. In addition, the white line intensity is in principle proportional to the *d* orbital density of states that are affected by the presence of multiple oxidation states and various electron configuration. The higher integrated white line intensity of Ir-Co_3_O_4_ indicates that it has more empty *d* orbital states (*d* holes) than IrO_2_, which may lead to enhanced electrocatalytic performances. Importantly, the information derived from Ir *L*_3_-edge extended X-ray absorption fine structure (EXAFS) of Ir-Co_3_O_4_ shows two distinct peaks in R space, in which the first peak can be clearly attributed to Ir-O bonds (Fig. 2e). The second bond cannot be distinguished directly due to the similar lengths of Ir-Ir bond (2.707 Å) and Ir-Co bond (2.91 Å) (discussed below). It was further recognized by the wavelet transform (WT)-EXAFS analysis, which exhibits two characteristic regions: a first shell domain for Ir-O scattering with a local maximum at R = 1.58 Å and k = 6.62 Å^−1^ and a second shell domain at R = 2.61 Å and k = 8.02 Å^−1^. Although the second shell domain in Ir-Co_3_O_4_ has a similar R value on that of Ir-Ir scattering in Ir foil (R = 2.65 Å and k = 10.04 Å^−1^), their k values are quite different, implying the existence of Ir-Co rather than Ir-Ir in the second shell scattering (Fig. 2f). Furthermore, a model-based EXAFS fit of Ir-Co_3_O_4_, IrO_2_ and Ir foil were performed and the results indicate that no Ir-Ir scattering can be discovered and that the second scattering can be attributed to Ir-Co with a distinct bond distance, which represents Ir-O-Co in the crystal structure (Supplementary Fig. 9 & Supplementary Table 3). Therefore, based upon of advanced electronic microscopy and spectroscopic techniques, we conclude that the Ir species exist as single atoms and are homogeneously dispersed in the Co_3_O_4_ host.

Unlike the XANES spectra at the 5*d*-*L*_3_ edge, where the strong white line represents an unoccupied 5*d* state, the dominate peak at the 3*d*-*K* edge represents an unoccupied 4*p* state, whose energy position and shape are determined primarily by the crystal structure. In such a case, the valence state of 3*d* elements can be confirmed by the energy position of the leading edge (at a normalized absorption position of 0.8)^48–50^. The sensitivity of the absorption edge to the valence state of 3*d* elements originates from a relatively large U_cd_ (1*s* core hole and 3*d* Coulomb interaction) as compared with U_dp_ (3*d* and 4*p* Coulomb interactions). As shown in Fig. 2g, the normalized Co *K*-edge XANES of Ir-Co_3_O_4_ clearly displays a slightly decreased valence state of the Co species induced by Ir incorporation. The corresponding Co *K*-edge EXAFS of Co_3_O_4_ and Ir-Co_3_O_4_ reveal three distinct peaks, identified as Co-O, octahedral Co-Co and tetrahedral Co-Co bonds, respectively^51,52^. However, a lower intensity of the Co-O and Co-Co bonds can be found in Ir-Co_3_O_4_ in comparison with the Co_3_O_4_, indicating higher atomic chaos caused by introducing Ir atoms (Fig. 2h). The WT-EXAFS analysis on Co *K*-edge is consistent with the EXAFS results (Supplementary Fig. 10).

### Electrochemical performance of Ir-Co_3_O_4_

**Fig. 3:**
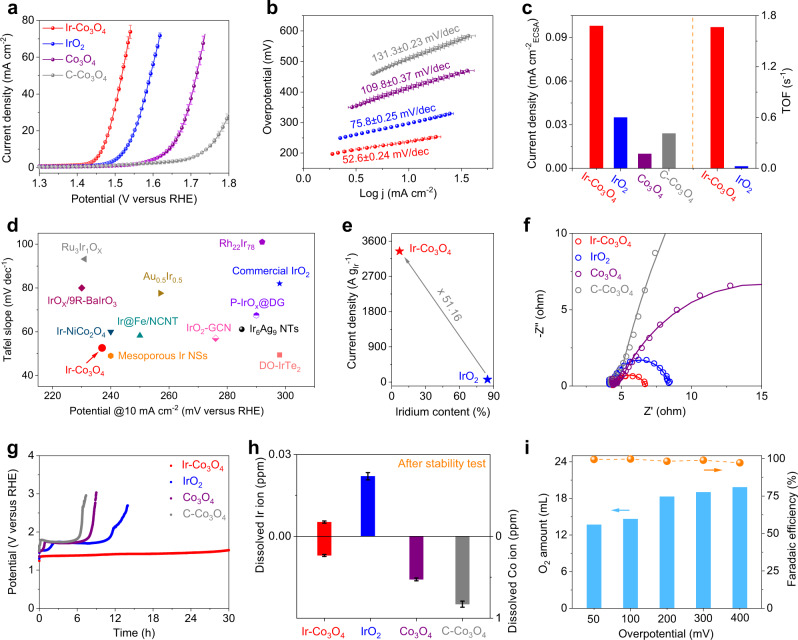
Electrochemical OER of Ir-Co_3_O_4_. **a** Polarization curves of Ir-Co_3_O_4_, IrO_2_, Co_3_O_4_ and C-Co_3_O_4_ in 0.5 M H_2_SO_4_ at a scanning rate of 5 mV s^−1^. **b** Tafel plots derived from the polarization curves in (**a**). **c** Corresponding specific activities of Ir-Co_3_O_4_, IrO_2_, Co_3_O_4_ and C-Co_3_O_4_; TOF values of Ir-Co_3_O_4_ and IrO_2_ at the overpotential of 300 mV. **d** Comparison of overpotentials and Tafel plots in reported Ir-based OER electrocatalysts. **e** Comparison of the mass activities normalized to Ir mass on Ir-Co_3_O_4_ and IrO_2_. **f** EIS Nyquist plots and fitting curves of samples recorded at the 1.43 V vs. RHE. **g** Chronopotentiometric measurements of Ir-Co_3_O_4_, IrO_2_, Co_3_O_4_ and C-Co_3_O_4_ at 10 mA cm^−2^, carbon paper was used as the catalyst support. **h** Dissolved Ir (left-y axis) and Co (right-y axis) ion concentrations measured for Ir-Co_3_O_4_, IrO_2_, Co_3_O_4_ and C-Co_3_O_4_ in the electrolyte by ICP-OES. **i** Generated O_2_ amount and corresponding Faradaic efficiency over a range of overpotentials on Ir-Co_3_O_4_. Note: error bars represent the standard deviation of three independent measurements.

The OER performances of Ir-Co_3_O_4_ and as-prepared Co_3_O_4_ in 0.5 M H_2_SO_4_ were investigated on glassy carbon electrode (GCE) together with the commercial IrO_2_ and commercial Co_3_O_4_ (denoted as IrO_2_ and C-Co_3_O_4_, respectively) for comparison (Supplementary Fig. 11). All measured potentials in this study were calibrated to the reversible hydrogen electrode (RHE) scale (Supplementary Fig. 12). As shown in Supplementary Fig. 13, Ir-Co_3_O_4_ exhibits a negatively shifted Co redox peak (III/IV) in the cyclic voltammetry (CV) curves compared to the Co_3_O_4_, indicating the isolated Ir sites can expedite the proton-electron transfer-mediated activation of Co to OER-active Co^4+^^53^. As expected, Ir-Co_3_O_4_ exhibits the best OER performances among all studied samples. In terms of reaching the current density of 10 mA cm^−2^, Ir-Co_3_O_4_ requires an overpotential (ƞ) of 236 mV, which is lower than that of Co_3_O_4_ (412 mV) and C-Co_3_O_4_ (511 mV). Notably, the low overpotential of Ir-Co_3_O_4_ even suppresses that of benchmark IrO_2_ (298 mV) with the same amounts of catalysts loading on electrode, as shown in Fig. 3a. Likewise, Ir-Co_3_O_4_ displays the lowest onset potential among these catalysts, showing that it has the fastest OER activation rate (Supplementary Fig. 14). The LSV curves of those catalysts without iR compensation are also displayed in Supplementary Fig. 15. In addition, the outstanding OER activity of Ir-Co_3_O_4_ is also reflected by the smallest Tafel slope of 52.6 mV dec^−1^ compared to that of IrO_2_ (75.8 mV dec^−1^), Co_3_O_4_ (109.8 mV dec^−1^) and C-Co_3_O_4_ (131.3 mV dec^−1^), indicating that the OER kinetic of Ir-Co_3_O_4_ is significantly accelerated (Fig. 3b). Corresponding graphs that plot the Tafel slope as a function of the overpotential on these catalysts are depicted in Supplementary Fig. 16, from which the boundary between kinetically-limited and mass transport limited currents can be clear distinguished. An obvious tendency of the boundary is followed as: Ir-Co_3_O_4_ < IrO_2_ < Co_3_O_4_ < C-Co_3_O_4_. The evaluation of an electrocatalyst system is generally assessed by two key factors: specific activity and turnover frequency (TOF). The electrochemical active surface area (ECSA) was firstly estimated from the double-layer capacitance (C_dl_) measurements as shown in Supplementary Fig. 17^54^. By normalizing the OER current to ECSA at ƞ = 300 mV, Ir-Co_3_O_4_ shows the highest specific activity (0.098 mA cm^−2^_ECSA_), almost 2.8 and 9.8 times higher than those of IrO_2_ (0.035 mA cm^−2^_ECSA_) and Co_3_O_4_ (0.01 mA cm^−2^_ECSA_). Moreover, Ir-Co_3_O_4_ delivers a high intrinsic activity of TOF value normalized to Ir sites per geometric area of 1.665 s^−1^, which is nearly 70-fold higher than that of IrO_2_ (0.0237 s^−1^) (Fig. 3c). Also, Ir-Co_3_O_4_ shows a higher TOF value than both Co_3_O_4_ and C-Co_3_O_4_ when normalized to Co sites per geometric area. In addition, ECSA-normalized TOF values further indicate the better intrinsic activity of Ir-Co_3_O_4_ (Supplementary Fig. 18). BET-normalized specific activities were calculated to exclude the morphology effects on activity assessment, in which Ir-Co_3_O_4_ also outperforms that of Co_3_O_4_ (Supplementary Fig. 19). The above results confirm the incorporated Ir single atoms can efficiently boost the catalytic performances of Co spinel oxide beyond that of the noble IrO_2_. A synopsis of previous literatures in Fig. 3d, highlights the merits of Ir-Co_3_O_4_, including low overpotential and Tafel slope are shown to be superior to most reported Ir-based OER electrocatalysts (Supplementary Table 4). In addition to the consideration of catalytic performance, cost-effectiveness is also of great significance for catalysts for practical applications. Thereafter the OER polarization currents of Ir-Co_3_O_4_ and commercial IrO_2_ were normalized by the loading mass of Ir at ƞ = 300 mV. In addition, the mass activities were calculated from the LSV on carbon paper electrodes with higher electrode surface areas (1 cm^2^) (Supplementary Fig. 20). As revealed in Fig. 3e, the mass activity of Ir-Co_3_O_4_ is 3343.37 A g^−1^_Ir_, which is 51.16 times higher than that of IrO_2_ (65.35 A g^−1^_Ir_) at the overpotential of 300 mV. The superb mass activity derived from each Ir atoms indicates the remarkable economic efficiency of Ir-Co_3_O_4_ towards OER. Electrochemical impedance spectroscopy (EIS) measurements of these four samples were tested under the same condition, as shown in Fig. 3f. In comparison with the charge transfer resistance (R_ct_) among IrO_2_ (4.18 Ω), Co_3_O_4_ (20.55 Ω) and C-Co_3_O_4_ (115.5 Ω), Ir-Co_3_O_4_ displays the smallest R_ct_ of 2.37 Ω by establishing the equivalent circuit and fitting the Nyquist plots, implying that it has the lowest charge transfer resistance and the fastest charge transfer rate for the OER reaction (Supplementary Fig. 21 & Supplementary Table 5)^55^.

In addition to the activity, durability is another crucial criterion for the electrocatalytic performance of catalysts towards OER under operating conditions. As plotted in Fig. 3g, Ir-Co_3_O_4_ shows long-term stability with a current density of 10 mA cm^−2^ for almost 30 h, which is superior to that of IrO_2_, Co_3_O_4_ and C-Co_3_O_4_ catalysts. Specifically, the commercial IrO_2_ and as-synthesized Co_3_O_4_ can only maintain the current densities for nearly 15 h and 9 h, respectively, before they deactivate. CV scanning experiments further highlight that Ir-Co_3_O_4_ exhibits a negligible increase of overpotential after 3000 cycles, whereas both Co_3_O_4_ and C-Co_3_O_4_ lost most of their activities (Supplementary Fig. 22). Both chronopotentiometric and CV scanning measurements demonstrate that the isolated Ir single sites enable activating the spinel Co oxide more durable in harsh OER environment. Further ICP-OES investigations show that leaching occurs preferentially at the Co species in Ir-Co_3_O_4_, but the dissolution effect of hosting Co_3_O_4_ is alleviated by dispersed Ir single sites during OER reaction. In contrast, IrO_2_, Co_3_O_4_ and C-Co_3_O_4_ suffer from severe Ir and Co ions leaching after the OER durability test (Fig. 3h & Supplementary Fig. 23)^56^. The enhanced stability of Ir-Co_3_O_4_ is induced by the doped effect of Ir single atoms, which is the main reason for the observed long-term stability in the acidic medium. In addition, the morphology characterizations by means of SEM and TEM reveal that Ir-Co_3_O_4_ preserves the original ultrathin nanosheets with slight fractures after the chronopotentiometry at 10 mA cm^−2^. No obvious structural changes can be found from the XRD pattern and HR-TEM images. Elemental mapping images show that Ir, Co and O elements are still homogeneously distributed over the entire Ir-Co_3_O_4_ sample after OER (Supplementary Fig. 24). However, both the Ir 4*f* and Co 2*p* XPS spectra of post-OER Ir-Co_3_O_4_ show increased valence states of Ir and Co, which is common for OER catalysts under oxidative voltages. The O 1*s* XPS binding energy of post-OER Ir-Co_3_O_4_ shifts to a more positive position, further confirming the increase of valence state on overall catalyst after the stability test (Supplementary Fig. 25). The stabilities of Ir-Co_3_O_4_ and IrO_2_ were further tested at a higher current density of 50 mA cm^−2^. As shown in Supplementary Fig. 26, Ir-Co_3_O_4_ maintains the long-term stability up to 6 h, while IrO_2_ loses its activity after only 2 hours, further indicating the activity retention of Ir-Co_3_O_4_ even at a high current density. On the other hand, complex OER is always accompanied with other side reactions such as catalyst/support oxidation, material dissolution and capacitive current effects, which may lead to the overestimation of actual activity and thus it is essential to estimate the associated OER Faradaic efficiency (FE) of the catalyst^57^. Gas chromatography (GC) was used to continuously monitor the generated O_2_ amount and calculate the FE on Ir-Co_3_O_4_ over a wide range of overpotentials (Supplementary Fig. 27). As depicted in Fig. 3i, the corresponding O_2_ production increases with the increasing OER overpotentials; the calculated FE for OER remains at almost 100% at small overpotentials but drops slightly to 97% for large overpotentials. The relatively high FE values signify that the majority of energy is applied to drive OER and almost no side reactions occur on Ir-Co_3_O_4_. Overall, the high electrode/intrinsic activity and electrochemical durability conjointly endow Ir-Co_3_O_4_ as a promising OER electrocatalyst candidate for practical applications in the acidic water electrolyzer, namely proton exchange membrane water electrolyzer. Going further, practical applications usually demand that the catalysts preparation process is contamination-free and non-toxic, as well as can easily realize gram-level preparation of catalysts in one batch^58^. Nevertheless, traditional synthesis methods for single-atom materials, including impregnation and solvothermal strategies usually require pollution-carrying organic solvent and their productions are seriously limited. For example, the similar atomic Ir-doped Co oxides have also been reported, but in which the multistep impregnation methods they employed can only prepare the electrocatalysts with milligram-level productions^59,60^. In contrast, the mechanochemical protocol we adopted for synthesizing Ir-Co_3_O_4_ is environmental-friendly, adding only NaCl and NaOH for preparation besides metal precursors; the production can be readily scaled up to 2.5 g per batch. Resulting SEM, TEM, HR-TEM images, XRD pattern and SEM-EDS spectrum reveal no obvious structural and compositional changes on the mass-produced Ir-Co_3_O_4_. Elemental mappings show that Ir, Co and O are homogeneously distributed over the Ir-Co_3_O_4_. In addition, this mass-produced sample can largely maintain its original OER activity, demonstrating the potential of Ir-Co_3_O_4_ for application in practical water electrolyzer (Supplementary Fig. 28).

### Mechanistic insights into OER process

**Fig. 4:**
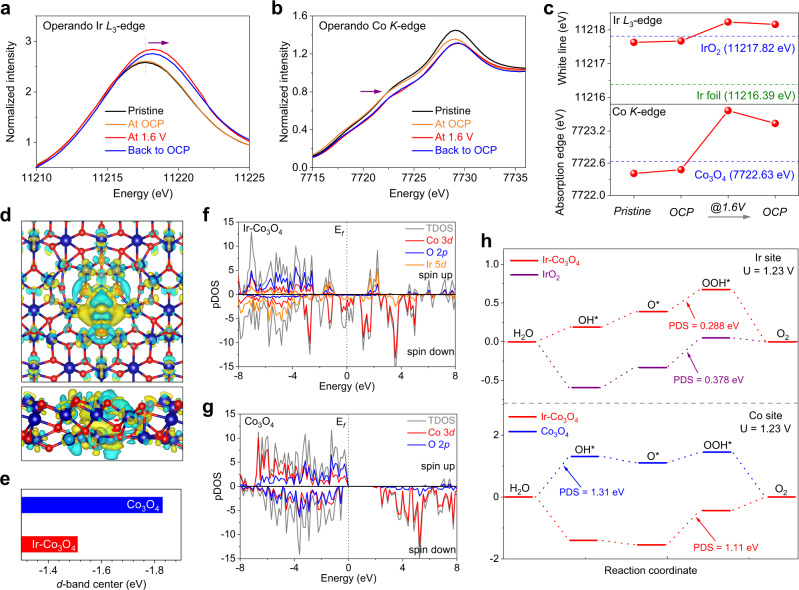
Operando XANES analysis and energetic properties of Ir-Co_3_O_4_ for OER. **a** The Ir *L*_3_-edge XANES and (**b**) the Co *K*-edge XANES of Ir-Co_3_O_4_ measured in 0.5 M H_2_SO_4_ under pristine state, OCP, OER operating condition of 1.6 V and OCP after OER operation. Ir *L*_3_-edge XANES of Ir foil and IrO_2_, and Co *K*-edge XANES of Co_3_O_4_ are used as references. **c** The white line positions and the absorption edge positions of all measured XANES. **d** The top and side panels of charge density difference on Ir-Co_3_O_4_. **e** Comparison of the *d*-band center of Ir-Co_3_O_4_ and Co_3_O_4_. Corresponding Co 3*d*, O 2*p* and Ir 5*d* pDOS spectra of (**f**) Ir-Co_3_O_4_ and (**g**) Co_3_O_4_. **h** The Gibbs free energy diagrams of the four-electron OER process on the Ir sites and Co sites of these catalysts under the applied overpotentials of 1.23 V vs. RHE, respectively.

Since the doped Ir single atoms promote the electrocatalytic performances of Co spinel oxide, the operando XANES tests at both Ir-*L*_3_ edge and Co *K*-edge were carried out on Ir-Co_3_O_4_ to learn more about the active sites and examine the chemical states evolution during the OER process. In the measurements, the operando XANES data were sequential collected under the open circuit potential (OCP), one representative potential (1.6 V vs. RHE) and then back to OCP again. Corresponding schematic diagram of the custom-built electrochemical cell and the obtained current-time (i-t) curves under applied voltages used for collecting the operando XAS data were depicted in Supplementary Fig. 29. As shown in Fig. 4a, the Ir-*L*_3_ edge white line position at OCP shows a slight positive shift in energy as compared with that of fresh Ir-Co_3_O_4_, probably resulting from the delocalization of electron caused by adsorbed H_2_O in the electrolyte. Upon increasing the applied voltage to 1.6 V, the peak position of Ir-*L*_3_ shifts higher in energy by 0.6 eV, which suggests that isolated Ir atoms are evidently oxidized with an increased valence state during OER. In addition, the oxidized Ir atoms retain high chemical states even when the applied potential is reversed to OCP, indicating that the valence change is irreversible on Ir-Co_3_O_4_ (Fig. 4c). As depicted in Supplementary Fig. 30, through further comparison of the white line position with standard references, it can be seen that the Ir-*L*_3_ energy peak of Ir-Co_3_O_4_ at the anodic potential of 1.6 V is located between the standard spectra of the Ir^4+^ reference La_2_CoIrO_6_ and the Ir^5+^ reference Sr_2_CoIrO_6_, confirming it has a chemical state between +4 and +5. The average state of Ir single atoms is further estimated to be +4.46 according to the absorption energy-valence state standard curve^26,48^. In other words, there is a transition from Ir^4+^ state to Ir^>4+^ state in part of the Ir-Co_3_O_4_ catalysts during the electrochemical reactions. The phenomena discovered by operando XAS is definitely meaningful, as demonstrated by previous research showing that the Ir species with high valence state in combination with electrophilic O ligands act as the active sites of OER^61,62^. It is believed that the formed covalent Ir-O sits and electrophilic O ligands promote the O-O formation via a nucleophilic attack and thus support the enhanced OER performances. This suggests that the isolated Ir atoms are efficiently activated, endowing Ir-Co_3_O_4_ with enhanced activity in OER^63–66^.

Subsequently, the valence change of Co species in Ir-Co_3_O_4_ was also probed by operando Co *K*-edge XANES (Fig. 4b). As shown in Fig. 4c, the Co-*K* absorption edge of Ir-Co_3_O_4_ at OCP is close to that of a Co_3_O_4_ reference, but it gradually shifts to a higher energy by 1.0 eV under an applied voltage of 1.6 V, indicating an increase in the Co valence state by 0.47 through comparison with the standard references of Co^2+^ reference (La_2_CoIrO_6_), Co^3+^ reference (EuCoO_3_) and Co^4+^ reference (BaCoO_3_) in Supplementary Fig. 31^48^. When the applied voltage was set back to OCP, the Co species in Ir-Co_3_O_4_ remain in the highly oxidized states. As a consequence, the continuously increased Ir and Co chemical states of Ir-Co_3_O_4_ under operating voltages demonstrates that the both the Ir single atoms and Co atoms are functionalized as the OER active sites in Ir-Co_3_O_4_, which are responsible for charge transfer in catalytic reactions together.

To further understand the OER mechanism and the synergistic function on Ir-Co_3_O_4_, a theoretical study was performed based on density functional theory (DFT) calculations. As shown in Fig. 4d, the charge density difference on Ir-Co_3_O_4_ indicates that the incorporation of Ir into Co_3_O_4_ can effectively regulate the charge redistribution, where the Ir atom tends to lose electrons and the delocalized electrons accumulate around adjacent Ir-O bonds. This result also confirms a strong electronic interaction between the Ir single site and the host Co_3_O_4_^67^. In addition, as shown in Fig. 4e it can be obtained that the *d*-band center position of Ir-Co_3_O_4_ (−1.512 eV) is higher than that of Co_3_O_4_ (−1.834 eV). The positive shift of *d*-band center would stabilize the interaction between the catalyst surface and the adsorbates, which is in strong alignment with the calculated free energy in Fig. 4h^68^. The projected density of states (pDOS) suggests that the introduction of Ir causes new hybridized electronic states in Ir-Co_3_O_4_, leading to a widening of the total density of states (TDOS) near the Fermi level, whereas the TDOS of Co_3_O_4_ displays a band gap. The apparent difference demonstrates that the introduction of Ir single atoms endow the Co_3_O_4_ with metallic characters, leading to a better electronic conductivity than that of Co_3_O_4_ for faster OER kinetics, which is consistent with our EIS measurements. Furthermore, the synergistic function between Ir atom and Co_3_O_4_ was investigated by checking the Co 3*d*, O 2*p* and Ir 5*d* pDOS, in which it can be clearly observed that the Ir 5*d* orbit is substantially overlapped with Co 2*p* orbit in pDOS, suggesting the strong interaction and more covalent character between Co and Ir atoms (Fig. 4f, g)^26^. In addition, the Gibbs free energy changes of acidic OER intermediates (OH*, O*, OOH*) in the elementary steps under the applied potential of 1.23 and 0 V vs. RHE are displayed in Fig. 4h and Supplementary Fig. 32, respectively. The potential determining steps (PDS) of Ir sites were first calculated and results reveal that the limiting steps occur at the conversion from O* to OOH* on both Ir-Co_3_O_4_ and IrO_2_. Their corresponding PDS values are 0.288 eV and 0.378 eV, suggesting the lower energy barrier and better OER performance of Ir-Co_3_O_4_. Furthermore, the calculations on Co sites indicate that the PDS on Co_3_O_4_ is primarily derived from the transformation from H_2_O to OH* with an energy barrier of 1.31 eV. However, the PDS is changed as the formation of OOH* and the corresponding energy barrier is further reduced to 1.11 eV on Ir-Co_3_O_4_, emphasizing the promoting effect toward OER derived from Ir single sites^29^. It is believed that the electrophilic characters render these oxygen ligands caused by high-valence Ir susceptible to nucleophilic acid-base-type O-O bond formation at reduced kinetic barrier, leading to enhanced OER activity^61,62^. Therefore, both the operando XAS characterizations and DFT calculations conjointly demonstrate that the remarkable catalytic performance on Ir-Co_3_O_4_ is due to the synergistic cooperation between Ir and Co single sites with their bridged O ligands as well as the distinctly reduced energy barrier toward OER.

## Discussion

In conclusion, the active Ir-Co_3_O_4_ OER catalyst was successfully synthesized via a facile and economical mechanochemical method. Both AC HAADF-STEM characterization and XAS technique confirm that isolated Ir single atoms are homogeneously dispersed in the Co_3_O_4_ host material. Consequently, OER measurement in acidic medium reveals that the overpotential of as-prepared Co_3_O_4_ (412 mV) sharply decreases to 236 mV by doping a trace amount of Ir atoms, and its long-term stability is prolonged. Notably, the normalized mass activity and TOF of Ir-Co_3_O_4_ are higher than those of commercial IrO_2_. By virtue of the operando XANES analysis, we recognize that the Ir single atoms are partially activated to higher valence under anodic voltages, simultaneously both Co and Ir atoms with their electrophilic O ligands served as active sites are synergistically responsible for charge transferring during OER process. DFT calculations further demonstrate the improved electronic conductivity and lower energy barrier towards OER by the electrophilic characters of oxygen ligands induced by high-valence Ir, boosting OER activity. Additionally, in contrast to most traditional synthesis methods for single-atom catalysts, which are limited to milligram-level production, the Ir-Co_3_O_4_ preparation can be easily scaled up to gram-level production with negligible activity loss. This study highlights the rational design of a promising, cost-effective and energy-efficient catalyst for boosting oxygen evolution in the acidic medium, and this prototype would potentially stimulate industrial interest in a larger scale for practical applications.

## Methods

### Materials

Iridium trichloride (IrCl_3_, solid, 99.9%) was purchased from Ark Pharm. Cobalt chloride hexahydrate (CoCl_2_.6H_2_O, solid, AR) was purchased from Aladdin. Sodium chloride (NaCl, solid, 99.8%) and sodium hydroxide (NaOH, solid, 97%) were purchased from Sinopharm Chemical Reagent Co. Ltd (Shanghai, China). Cobalt oxide (Co_3_O_4_, solid, 99.7%) was purchased from Alfa Aesar. Iridium oxide (IrO_2_, solid, 99.9%) was purchased from Innochem. Isopropanol (C_3_H_8_O, liquid, ≥ 99.7%) and sulfuric acid (H_2_SO_4_, liquid, ≥ 96%) were purchased from Sinopharm Chemical Reagent Co. Ltd (Shanghai, China).

### Catalyst synthesis

The preparation of Ir-Co_3_O_4_ was based on mechanochemical method. In a typical synthesis, 475.9 mg CoCl_2_.6H_2_O (2 mmol) and 29.9 mg IrCl_3_ (0.1 mmol) were initially mixed in agate mortar for 5 min. Afterwards, 0.5 g NaCl was added into the evenly mixed precursors and ground for 10 min. A homogeneous powder was obtained and then put into a high-energy milling pot (Planetary Mono Mill, Pulverisette 6, Fritsch) with stainless steel balls for grinding for 1 h under a rotation speed of 500 rpm. After the first milling, 1 mL NaOH solution (5 mmol mL^−1^) was added into the above mixture and ball milling was continued for 1 h. Finally, the obtained slurry was collected from the milling pot and dried at 60 °C in an oven under air atmosphere, then it was calcined in air at 350 °C for 6 h with a heating rate of 5 °C min^−1^. After calcination, the sample was washed by water 3 times and dried overnight at 80 °C. The as-synthesized Co_3_O_4_ was prepared by a similar method as Ir-Co_3_O_4_, except that IrCl_3_ was not added as a precursor.

### Scale-up synthesis

To realize the mass production of Ir-Co_3_O_4_, 2855.4 mg CoCl_2_.6H_2_O (12 mmol) and 179.4 mg IrCl_3_ (0.6 mmol) were initially mixed in agate mortar for 20 min. Afterwards, 3.0 g NaCl was added into the evenly mixed precursors and ground for 20 min. The homogeneous powder was obtained and then put into the high-energy milling pot with stainless steel balls and ground for 3 h at a rotation speed of 500 rpm. After the first milling, 6 mL NaOH solution (5 mmol L^−1^) was added into the above mixture and ball milling was continued for 3 h. Finally, the obtained slurry was collected from the milling pot and then calcined in air at 350 °C for 6 h at a heating rate of 5 °C s^−1^. After calcination, the sample was washed by water 3 times and dried overnight at 80 °C.

### Characterization

Scanning electron microscopy (SEM) images were collected on a Zeiss Supra 55 at an acceleration voltage of 5 kV. Transmission electron microscopy (TEM) and High-resolution TEM (HR-TEM) images were determined by a JEOL 2100F instrument at a working voltage of 200 kV. The aberration-corrected high angle annular dark field-scanning TEM (AC HAADF-STEM) images and corresponding elemental mapping were collected on an aberration correction Hitachi 2700D microscope operated at 200 kV. The N_2_ absorption/desorption isotherms were tested on a Autosorb iQ Quantachrome device and the specific surface area with pore distribution were obtained by the Brunauer–Emmett-Teller (BET) analysis. The mass fraction of Ir in Ir-Co_3_O_4_ and the dissolved ion concentrations after stability tests were determined by ICP-OES on an Agilent ICP-OES 730. The amount of O_2_ generated over a range of overpotentials were recorded by Agilent 7890B equipped with a 5 Å molecular sieve column thermal conductivity detector; Ar was used as the carrier gas.

The electronic structure was investigated by XPS on a Thermo Scientific Escalab 250Xi with an Al-K_α_ source. XAS of Co *K*-edge and Ir *L*_3_-edge were carried out at the 17 C beamline at the National Synchrotron Radiation Research Center (NSRRC) in Taiwan. The operando XAS experiments of Co *K*-edge and Ir *L*_3_-edge were performed at the 17 C beamline at the NSRRC with a custom-built operando XAS instruments, corresponding data was collected by applying the constant voltage on the catalyst and the electrochemical bias was controlled as 1.6 V vs. RHE. All XAS experiments were carried out in ambient air under room temperature and analyzed using the standard program Demeter. For wavelet-transformed *k*^3^-weighted EXAFS, the χ(k) exported from Athena was imported into the Hama Fortran code designed by Harald Funke and Marina Chukalina. The parameters were listed as follows: R range, 0-6 Å, k range, 0-15 Å^−1^; k weight, 0; and Morlet function with kappaMorlet = 10, sigmaMorlet = 1 was used as the mother wavelet to provide the overall distribution.

The structure was analyzed by XRD with a Bruker D8 Advance powder diffractometer (operating at 40 kV, 40 mA) equipped with a Cu-K_α_ source (*λ*1 = 1.5405 Å, *λ*2 = 1.5443 Å) and fitted with a beryllium window at room temperature. Rietveld refinements for XRD data were carried out with the Full-Prof program, and the refined parameters were background parameters, line shift errors (zero shift), Caglioti coefficients (U, V and W), scale factor, lattice parameters, atomic position, atomic rate occupancy and isotropic atomic displacement parameters.

### Electrochemical measurement

The OER activity measurements in O_2_-saturated 0.5 M H_2_SO_4_ were performed at room temperature on a CHI 760E electrochemistry workstation with a rotating disk electrode (RDE) configuration (Pine Research Instrumentation) via using a standard three-electrode electrochemical cell. A glassy carbon (GC) electrode (0.196 cm^2^), calomel electrode and graphite rod were used as the working, reference and counter electrode, respectively. Prior to each testing, the working electrode was ground and polished three times by fine aluminum oxide powder and washed with distilled water to remove the residual catalyst. The counter electrode was also cleaned by distilled water before being used. Before the electrocatalytic tests, samples (5 mg), carbon black (5 mg, Vulcan XC-72R) and 5 wt% Nafion solution (0.1 mL, Aldrich) were dispersed in absolute isopropanol (1.9 mL) via mild sonication to produce a homogeneous catalysts ink. Then, 20 μL of the catalyst ink was dropped onto the GC electrode with a diameter of 0.5 cm and dried under infrared light. The active materials that dropped on the electrode were 50 wt%. Metal mass loadings on the electrode for all samples were controlled at 0.255 mg cm^−2^. The Ir mass loadings for Ir-Co_3_O_4_ and IrO_2_ were calculated to be 0.018 mg cm^−2^ and 0.218 mg cm^−2^, respectively. The Co mass loadings for Ir-Co_3_O_4_, Co_3_O_4_ and C-Co_3_O_4_ were calculated to be 0.174 mg cm^−2^, 0.187 mg cm^−2^ and 0.187 mg cm^−2^, respectively. All OER potentials were calibrated with respect to RHE scale (Supplementary Fig. 12). The calibration was performed by using a Pt wire as the working electrode, the calomel electrode as the reference electrode and a graphite rod as the counter electrode in a H_2_-saturated electrolyte (0.5 M H_2_SO_4_). Linear sweep voltammetry (LSV) was carried out with a scan rate of 1 mV s^−1^ and the potential at which the current crossed zero was the calibration value. Cyclic voltammetry (CV) scans were initially performed at the scan rate of 100 mV s^−1^ between 1.1 and 1.5 V (vs. RHE) with no iR compensation for 50 cycles to activate and clean the catalyst surfaces. Subsequently, LSV with *i*R correction was conducted at a scan rate of 5 mV s^−1^ under room temperature to test the OER activity. The *i*R correction was automatic compensation by the used electrochemical workstation and was controlled as 95% rather than 100% to prevent the resonance and large data error. Tafel slopes were probed on Ir-Co_3_O_4_, IrO_2_, Co_3_O_4_ and C-Co_3_O_4_ within the potential range of 1.43–1.48 V vs. RHE, 1.48–1.56 V vs. RHE, 1.58–1.7 V vs. RHE and 1.69–1.81 V vs. RHE, respectively. And the Tafel slopes were determined by plotting the overpotential vs. the logarithm of current density (log |j|). Stability was evaluated using catalysts loaded carbon paper (1 cm^2^) with mass loadings of 0.255 mg cm^−2^ to carry out chronopotentiometry at a constant OER current density of 10 mA cm^−2^. Cyclic stability tests on Ir-Co_3_O_4_, IrO_2_, Co_3_O_4_ and C-Co_3_O_4_ were performed under room temperature within the potential range of 1.1–1.5 V vs. RHE, 1.16–1.56 V vs. RHE, 1.28–1.68 V vs. RHE and 1.38–1.78 V vs. RHE for 3000 cycles, respectively. All scan rates were controlled as 100 mV s^−1^. Electrochemical impedance spectroscopy was measured on the Bio-Logic SP-200 workstation in the frequency range of 0.01–100 kHz at the 1.43 V vs. RHE; a corresponding equivalent circuit and fitting resistance value were analyzed by EC-lab Software Analysis.

### Calculation of the electrochemically surface area

The electrochemically active surface area (ECSA) for also catalysts was estimated from the electrochemical double-layer capacitance (C_dl_) of the catalytic surface according to Eq. (1):

1 $${{{{{\rm{ECSA}}}}}}={R}_{f}S=\frac{{C}_{{dl}}}{{C}_{s}}S$$

where C_dl_ was measured from the scan-rate-dependent CVs in the non-Faradaic region of 0.75–0.95 V vs. RHE in 0.5 M H_2_SO_4_ with scan rates of 20, 40, 60, 80 and 100 mV s^−1^. The units for R_f_ and S are cm^2^_real_ cm^−2^_geo_ and cm^2^_geo_, respectively. S stands for the real surface area of the smooth metal electrode, which was generally equal to the geometric area of the glassy carbon electrode (S = 0.196 cm^2^). The specific capacitance (C_s_) for a flat surface was found to be in the range of 20-60 μF cm^−2^. In this work, a C_s_ value of 60 μF cm^−2^ was used to estimate the ECSA.

### Calculation of the turnover frequency

The turnover frequency (TOF) of these catalysts was calculated using Eq. (2):

2 $${{{{{\rm{TOF}}}}}}=\frac{\#{{{{{\rm{number}}}}}}\; {{{{{\rm{of}}}}}}\; {{{{{\rm{total}}}}}}\; {{{{{\rm{oxygen}}}}}}\; {{{{{\rm{turenover}}}}}}/{{{{{{\rm{cm}}}}}}}_{{{{{{\rm{geo}}}}}}}^{2}}{\#{{{{{\rm{{number}}}}}}}\;{{{{{\rm{of}}}}}}\;{{{{{\rm{active}}}}}}\; {{{{{\rm{sites}}}}}}/{{{{{{\rm{cm}}}}}}}_{{{{{{\rm{geo}}}}}}}^{2}}$$

the O_2_ turnover per geometric area was obtained from the geometric current density for the LSV curves according to Eq. (3):

3 $${{{{{{\rm{O}}}}}}}_{2}\,{{{{{\rm{turnover}}}}}}\;{{{{{\rm{per}}}}}}\;{{{{{\rm{geometric}}}}}}\;{{{{{\rm{area}}}}}}=	 {{{{{{\rm{j}}}}}}}_{{{{{{\rm{geo}}}}}}} \times \frac{1\,{{{{{\rm{C}}}}}}{{{{{{\rm{s}}}}}}}^{-1}}{1000\,{{{{{\rm{mA}}}}}}} \times \frac{1\,{{{{{\rm{mol}}}}}}}{96485.3\,{{{{{\rm{C}}}}}}}\\ 	 \times \frac{1}{4} \times \frac{6.02\times {10}^{23}}{1\,{{{{{\rm{mol}}}}}} \, {{{{{{\rm{O}}}}}}}_{2}}$$

the number of the active sites per geometric area can be calculated from the results of the ICP-AES analysis. For example, the upper limit of Ir site density of Ir-Co_3_O_4_ is:

$$0.05{{{{{\rm{mg}}}}}} \times 6.9\% \times \frac{1{{{{{\rm{mmol}}}}}}}{192.2{{{{{\rm{mg}}}}}}} \times \frac{6.02 \times {10}^{20}{{{{{\rm{sites}}}}}}}{1{{{{{\rm{mmol}}}}}}} \times \frac{1}{0.196\,{{{{{{\rm{cm}}}}}}}^{2}}=5.51 \times {10}^{16}\frac{{{{{{\rm{sites}}}}}}}{{{{{{{\rm{cm}}}}}}}^{2}}$$

At the overpotential of 300 mV, the OER current density for Ir-Co_3_O_4_ is 58.84 mA cm^−2^. And the corresponding TOF value normalized to geometric area was calculated to be:

$${{{{{{\rm{TOF}}}}}}}_{{{{{{\rm{geo}}}}}}}=\frac{1.56 \times {10}^{15}\frac{{{{{{{\rm{O}}}}}}}_{2}}{{{{{{{\rm{cm}}}}}}}^{2}}\,{{{{{{\rm{s}}}}}}}^{-1}{{{{{\rm{per}}}}}}\frac{{{{{{\rm{mA}}}}}}}{{{{{{{\rm{cm}}}}}}}^{2}}\times 58.84\frac{{{{{{\rm{mA}}}}}}}{{{{{{{\rm{cm}}}}}}}^{2}}}{5.51\times {10}^{16}\frac{{{{{{\rm{site}}}}}}}{{{{{{{\rm{cm}}}}}}}^{2}}}=1.665\,{{{{{{\rm{s}}}}}}}^{-1}$$

In addition, the O_2_ turnover per ECSA was obtained according to Eq. (4):

4 $${{{{{{\rm{TOF}}}}}}}_{{{{{{\rm{ECSA}}}}}}}=\frac{{{\#}} {{{{{\rm{number}}}}}}\; {{{{{\rm{of}}}}}}\; {{{{{\rm{total}}}}}}\; {{{{{\rm{oxygen}}}}}}\; {{{{{\rm{turenover}}}}}}\times {{{{{\rm{absoult}}}}}}\; {{{{{\rm{value}}}}}}\; {{{{{\rm{of}}}}}}\; {{{{{\rm{current}}}}}}\; {{{{{\rm{density}}}}}}}{\#{{{{{\rm{active}}}}}}\; {{{{{\rm{sites}}}}}} \times {{{{{\rm{ECSA}}}}}}}$$

Thus the corresponding TOF value of Ir-Co_3_O_4_ normalized to ECSA polarization information was calculated to be:

$${{{{{{\rm{TOF}}}}}}}_{{{{{{\rm{ECSA}}}}}}}=\frac{1.56\times {10}^{15}\frac{{{{{{{\rm{O}}}}}}}_{2}}{{{{{{{\rm{cm}}}}}}}^{2}}\,{{{{{{\rm{s}}}}}}}^{-1}{{{{{\rm{per}}}}}}\frac{{{{{{\rm{mA}}}}}}}{{{{{{{\rm{cm}}}}}}}^{2}}\times 58.84\frac{{{{{{\rm{mA}}}}}}}{{{{{{{\rm{cm}}}}}}}^{2}}}{5.51\times {10}^{16}\frac{{{{{{\rm{site}}}}}}}{{{{{{{\rm{cm}}}}}}}^{2}}\times 125.9\,{{{{{{\rm{cm}}}}}}}^{2}}=0.0132\,{{{{{{\rm{s}}}}}}}^{-1}$$

The TOF per metal site is calculated based hypothesis that all Ir or all Co atoms are accessible to the electrolyte in all cases. Other catalysts were calculated according to the same procedure.

### Calculation of OER Faradaic efficiency

The amount of produced O_2_ was tested for Ir-Co_3_O_4_ dropped carbon paper with a mass loading of 0.255 mg cm^−2^ as the working electrode to conduct OER in a sealed cell. The applied potentials on sample to generated O_2_ were 1.28 V, 1.33 V, 1.43 V, 1.53 V and 1.63 V vs. RHE, respectively. Then Ar was used as the carrier gas to purge the produced O_2_ into a gas chromatograph (Agilent 7890B) equipped with a 5 Å molecular sieve column thermal conductivity detector to generate the O_2_ signal peak. By fitting the relationship between a certain amount of O_2_ and the corresponding GC O_2_ signal area, a standard curve was established. By transforming the generated O_2_ peak area at different overpotentials into the standard curve, the generated O_2_ amounts on Ir-Co_3_O_4_ were calculated. The OER Faradaic efficiency (FE) of Ir-Co_3_O_4_ was obtained according to:

5 $${{{{{\rm{FE}}}}}}=\frac{{neF}}{Q}$$

where e is the number of electrons transferred for generating O_2_, Q is the total charge, n is the amount of generated O_2_ (in moles) and F is the Faradaic constant.

### Computational details

The geometries and energies were performed by the density functional theory (DFT) calculations with the Vienna Ab-initio Simulation Package (VASP)^69,70^. The interactions between ion cores and valence electrons were described by projector augmented wave (PAW) method^71^. The exchange correlation interaction was described with the generalized gradient approximation (GGA) with the Perdew-Burke-Ernzerhof (PBE) functional^72^. Dispersion correction (DFT-D3) was considered in all energy-related calculations. The wave functions at each k-point were expanded with a plane wave basis set with a kinetic cutoff energy of 400 eV. Geometries were optimized using a force-based conjugate-gradient method until the energy was converged to 1.0 × 10^−7^ eV/atom and the force to 0.02 eV/Å. The Brillouin zone was sampled with a 3 × 4 × 1 Monkhorst−Pack k-point mesh. The optimized (111) plane was adopted as the model for surface reaction pathways. Co_3_O_4_ (111) was constructed by cutting the bulk Co_3_O_4_ alone 111 directions. Ir doped Co_3_O_4_ was prepared by replacing one Co atom on the surface. During the optimization the up-layer atoms were allowed to relax and the atoms in the bottom layer were fixed to match the bulk structure. A vacuum layer of 15 Å was used along the c direction normal to the surface to avoid periodic interactions. The pDOS calculations were taken from the bare surfaces of studied samples and were calculated by hybrid functional HSE06 for the accuracy of electron distribution^73^. The *d*-band center was extracted from the average of the integral of *d*-band pDOS information. The Gibbs free energy was calculated based on the four-electron OER mechanism proposed by Nørskov^16^, corresponding formulas are listed as:

6 $$\begin{array}{c}2{{{{{{\rm{H}}}}}}}_{2}{{{{{\rm{O}}}}}}\,({{{{{\rm{l}}}}}})+\ast \to {{{{{{\rm{H}}}}}}}_{2}{{{{{\rm{O}}}}}}+{{{{{\rm{HO}}}}}}\ast+{{{{{{\rm{H}}}}}}}^{+}+{{{{{{\rm{e}}}}}}}^{-}\\ \varDelta {G}_{1}=\varDelta {{{{{{\rm{G}}}}}}}_{{{{{{\rm{HO}}}}}}}-\varDelta {{{{{{\rm{G}}}}}}}_{{{{{{\rm{H}}}}}}2{{{{{\rm{O}}}}}}}-{{{{{\rm{eU}}}}}}\end{array}$$

7 $$\begin{array}{c}{{{{{{\rm{H}}}}}}}_{2}{{{{{\rm{O}}}}}}\,({{{{{\rm{l}}}}}})+{{{{{\rm{HO}}}}}}\ast+{{{{{\rm{e}}}}}}^{-}+{{{{{{\rm{H}}}}}}}^{+}\to {{{{{{\rm{H}}}}}}}_{2}{{{{{\rm{O}}}}}}+{{{{{\rm{O}}}}}}\ast+2({{{{{{\rm{H}}}}}}}^{+}+{{{{{{\rm{e}}}}}}}^{-})\\ \varDelta {{{{{{\rm{G}}}}}}}_{2}=\varDelta {{{{{{\rm{G}}}}}}}_{{{{{{\rm{O}}}}}}}-{{{{{{\rm{DG}}}}}}}_{{{{{{\rm{HO}}}}}}}{-}{{{{{\rm{eU}}}}}}\end{array}$$

8 $$\begin{array}{c}{{{{{{\rm{H}}}}}}}_{2}{{{{{\rm{O}}}}}}\,({{{{{\rm{l}}}}}})+{{{{{\rm{O}}}}}}\ast+2({{{{{{\rm{H}}}}}}}^{+}+{{{{{{\rm{e}}}}}}}^{-})\to {{{{{\rm{HOO}}}}}}\ast+3({{{{{{\rm{H}}}}}}}^{+}+{{{{{{\rm{e}}}}}}}^{-})\\ \varDelta {{{{{{\rm{G}}}}}}}_{3}=\varDelta {{{{{{\rm{G}}}}}}}_{{{{{{\rm{HOO}}}}}}}-\varDelta {{{{{{\rm{G}}}}}}}_{{{{{{\rm{O}}}}}}}{-}{{{{{\rm{eU}}}}}}\end{array}$$

9 $$\begin{array}{c}{{{{{\rm{HOO}}}}}}\,\ast+3({{{{{{\rm{H}}}}}}}^{+}+{{{{{{\rm{e}}}}}}}^{-})\to {{{{{{\rm{O}}}}}}}_{2}({{{{{\rm{g}}}}}})+\ast+4({{{{{{\rm{H}}}}}}}^{+}+{{{{{{\rm{e}}}}}}}^{-})\\ \varDelta {{{{{{\rm{G}}}}}}}_{4}=\varDelta {{{{{{\rm{G}}}}}}}_{{{{{{\rm{O}}}}}}2}-\varDelta {{{{{{\rm{G}}}}}}}_{{{{{{\rm{HOO}}}}}}}-{{{{{\rm{eU}}}}}}\end{array}$$

Both free energy profiles were calculated on the Ir and Co sites in samples, respectively. The applied overpotentials were set as 0 and 1.23 V vs. RHE, respectively. Free energy diagrams were estimated as ΔG = ΔE + ΔZPE - TΔS, where E, ZPE, T, and S are the total energy obtained from DFT calculations, the zero-point energy (ZPE), the temperature (298.15 K), the entropy obtained from vibrational frequency calculations, respectively^74,75^. In addition, the frequencies were set to be 50 cm^−1^.

### Supplementary information

Supplementary Information

### Source data

Source Data

## Data Availability

All relevant data are available from the corresponding authors on request. Source data are provided with this paper.
