# Author Correction: Iridium single atoms incorporated in Co_3_O_4_ efficiently catalyze the oxygen evolution in acidic conditions

**DOI:** 10.1038/s41467-024-45791-1

**Published:** 2024-02-15

**Authors:** Yiming Zhu, Jiaao Wang, Toshinari Koketsu, Matthias Kroschel, Jin-Ming Chen, Su-Yang Hsu, Graeme Henkelman, Zhiwei Hu, Peter Strasser, Jiwei Ma

**Affiliations:** 1 Shanghai Key Laboratory for R&D and Application of Metallic Functional Materials, Institute of New Energy for Vehicles, School of Materials Science and Engineering, Tongji University, Shanghai, 201804 China; 2 Department of Chemistry and the Oden Institute for Computational Engineering and Sciences, The University of Texas at Austin, Austin, TX 78712-0165 USA; 3 Department of Chemistry, Technical University Berlin, 10623 Berlin, Germany; 4 National Synchrotron Radiation Research Center, Hsinchu, 30076 Taiwan; 5 Max Planck Institute for Chemical Physics of Solids, Nöthnitzer Strasse 40, 01187 Dresden, Germany

**Keywords:** Nanoscale materials, Electrocatalysis, Electrocatalysis

Correction to: *Nature Communications* 10.1038/s41467-022-35426-8, published online 14 December 2022

The original version of this Article contained an error in the ‘Catalyst synthesis’ method section. The 1 mL NaOH solution was mistakenly given as 5 mmol L^−1^ and heating rate as 5 °C s^−1^. These are now corrected to 5 mmol mL^−1^ and 5 °C min^−1^. This has been corrected in both the PDF and HTML versions of the Article.

