# Supplementary material for: Iridium single atoms incorporated in Co3O4 efficiently catalyze the oxygen evolution in acidic conditions
Source: Nat Commun. 2022 Dec 14;13:7754. doi: 10.1038/s41467-022-35426-8 (PMC9751110; doi:10.1038/s41467-022-35426-8)
Supplement: Supplementary file 1 — Supplementary Information [file 41467_2022_35426_MOESM1_ESM.pdf]

Supporting Information for

Iridium single atoms incorporated in Co_3O_4 efficiently catalyze the oxygen evolution in acidic conditions

Zhu *et al.*

Supplementary Figures

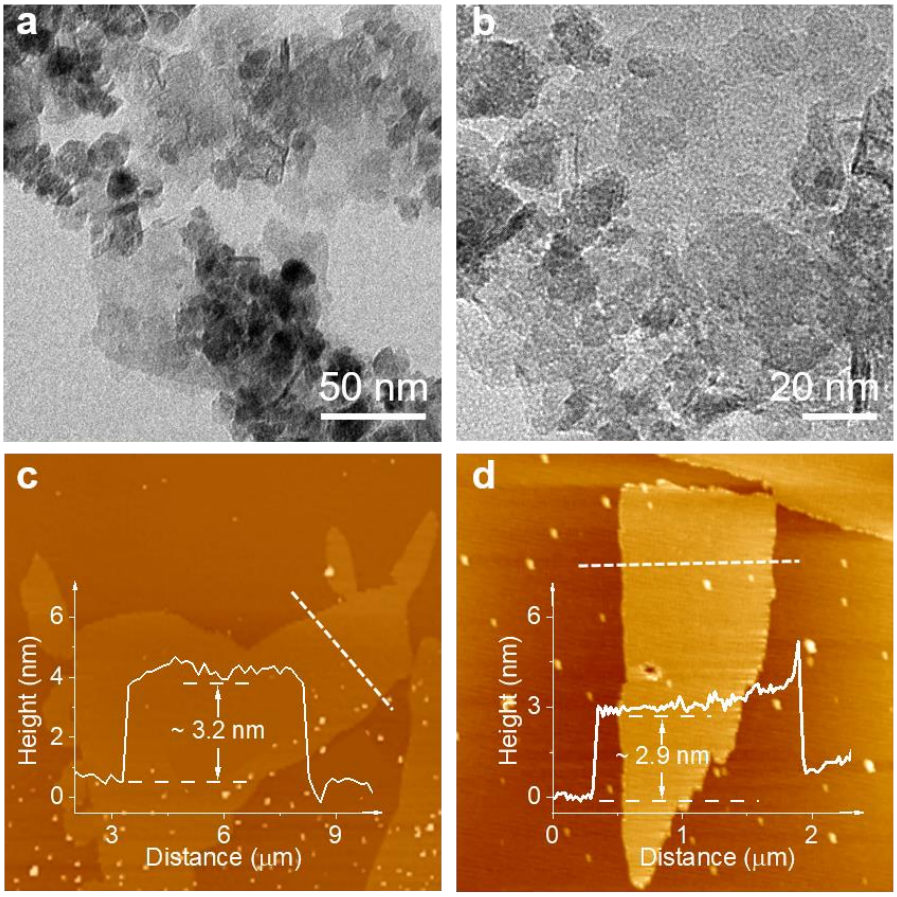

Supplementary Figure 1. Additional (a, b) TEM images and (c, d) AFM images for Ir-Co_3O_4.

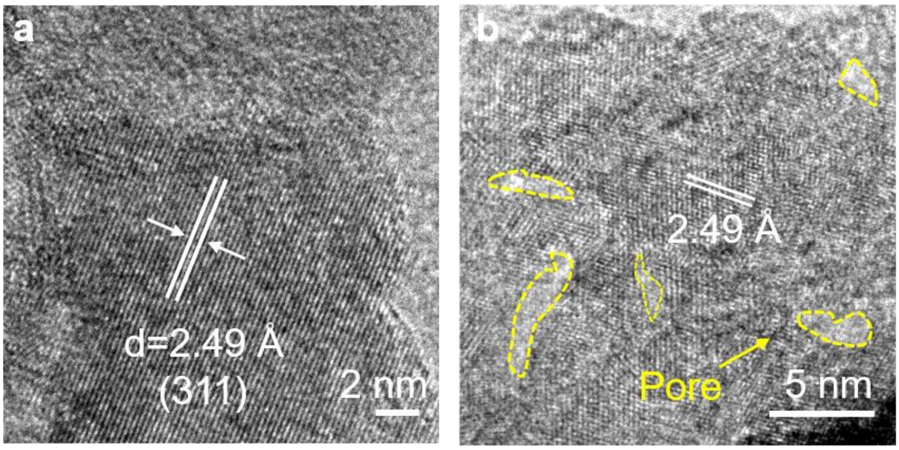

Supplementary Figure 2. HRTEM images of Ir-Co_3O_4.Yellow circles represent the defective pores on Ir-Co_3O_4.

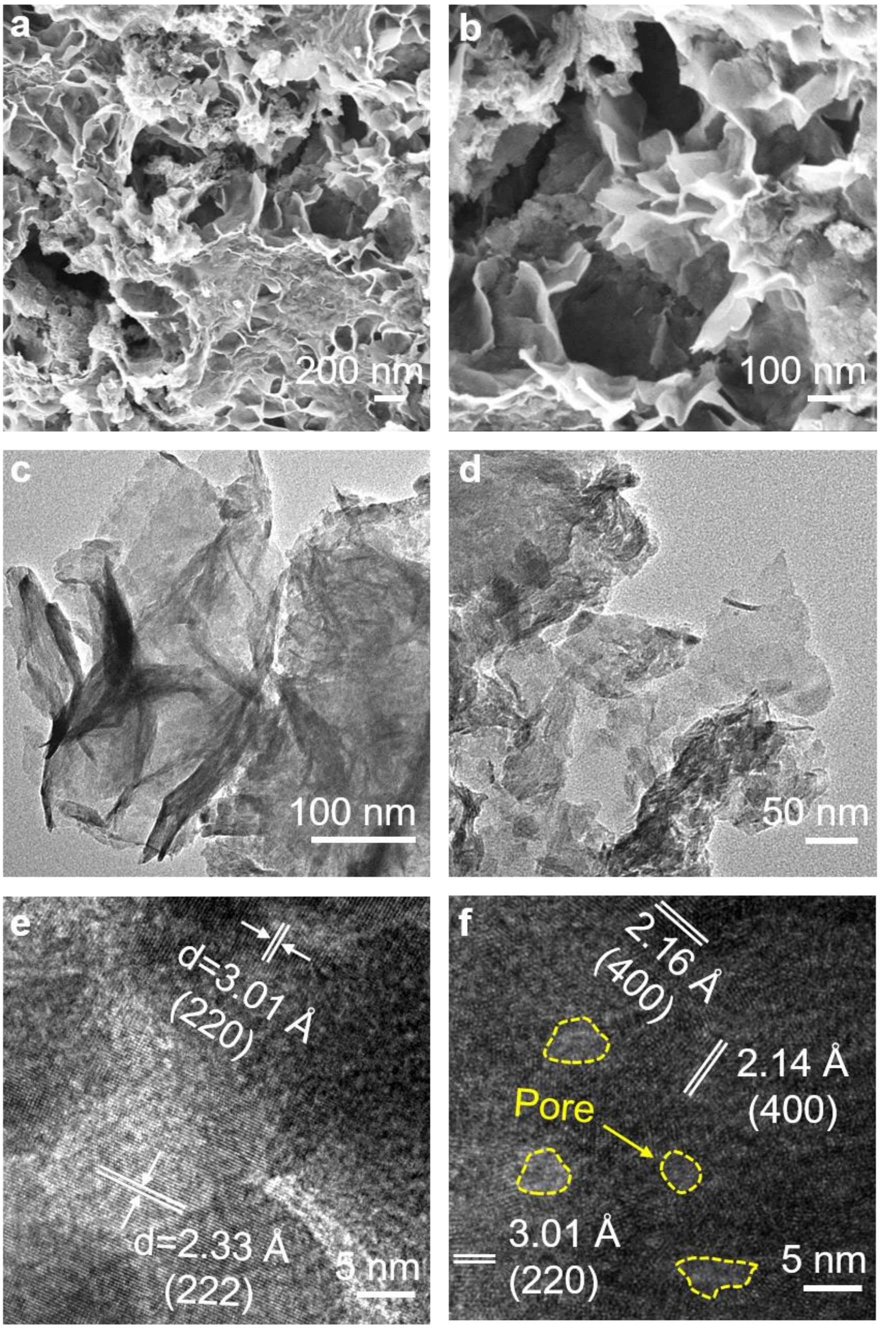

Supplementary Figure 3. (a, b) SEM images, (c, d) TEM images and (e, f) HRTEM images of as-synthesized Co_3O_4. Yellow circles represent the defective pores on Co_3O_4.

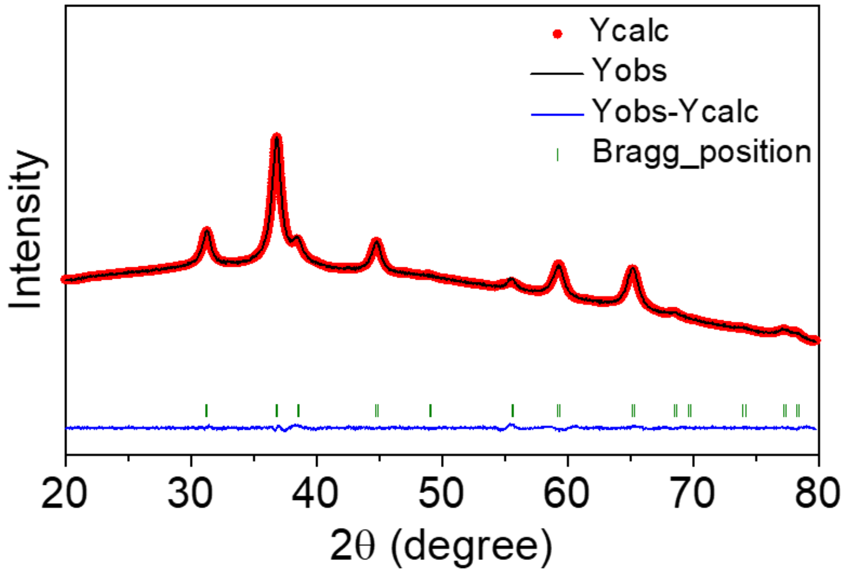

Supplementary Figure 4. Rietveld refinements of XRD patterns on Ir-Co_3O_4.

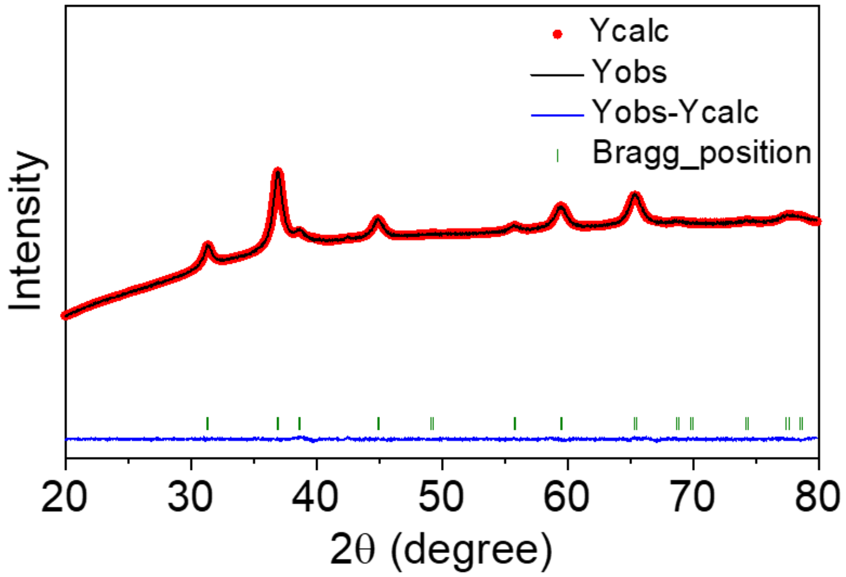

Supplementary Figure 5. Rietveld refinements of XRD patterns on as-synthesized Co_3O_4.

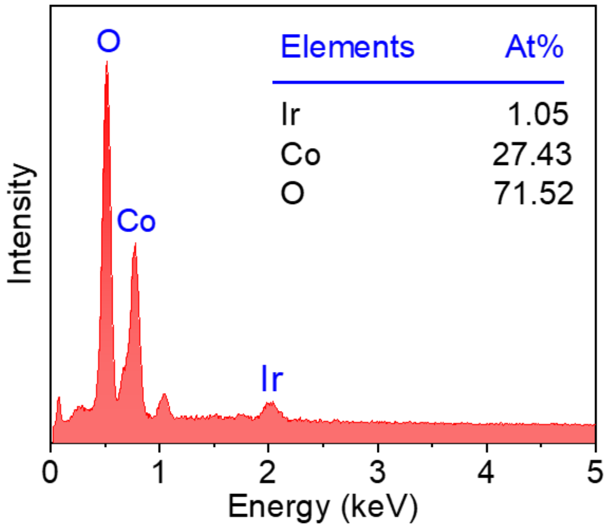

Supplementary Figure 6. SEM-EDS spectrum of Ir-Co_3O_4.

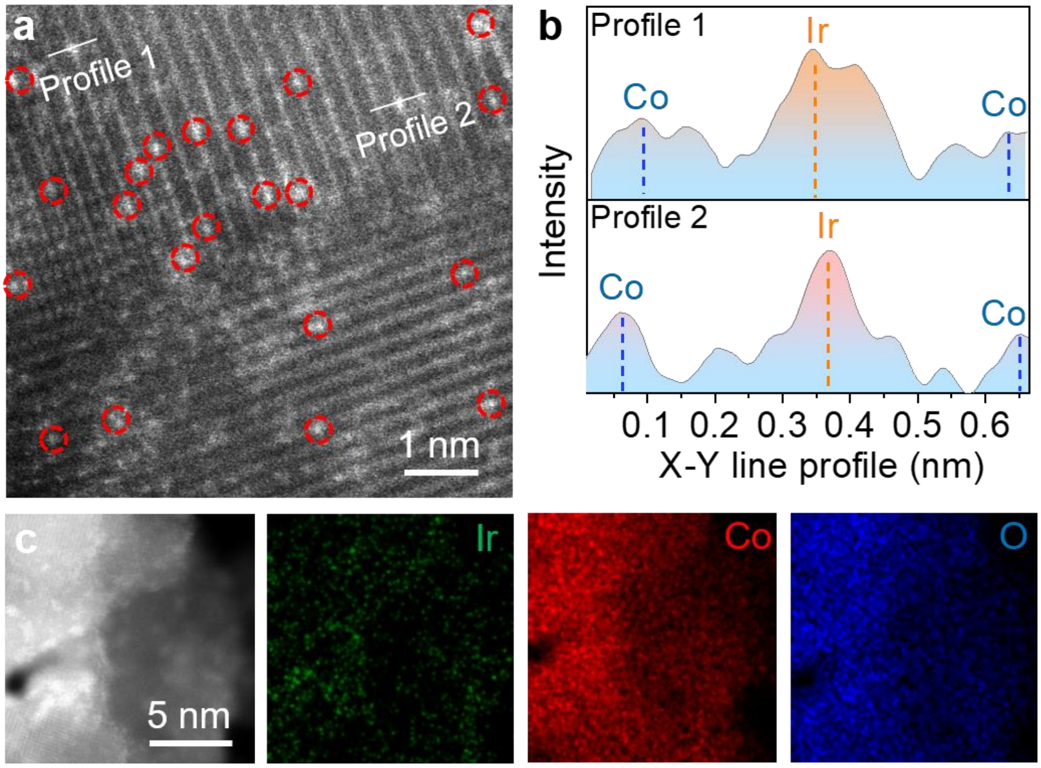

Supplementary Figure 7. (a) AC HAADF-STEM image of Ir-Co_3O_4. (b) The intensity profiles along the dash lines in (a). (c) AC HAADF-STEM image and the corresponding elemental mappings of Ir-Co_3O_4.

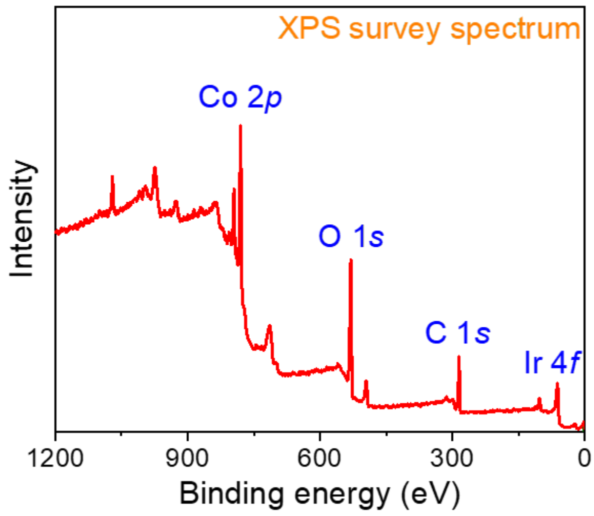

Supplementary Figure 8. XPS survey spectrum of Ir-Co_3O_4.

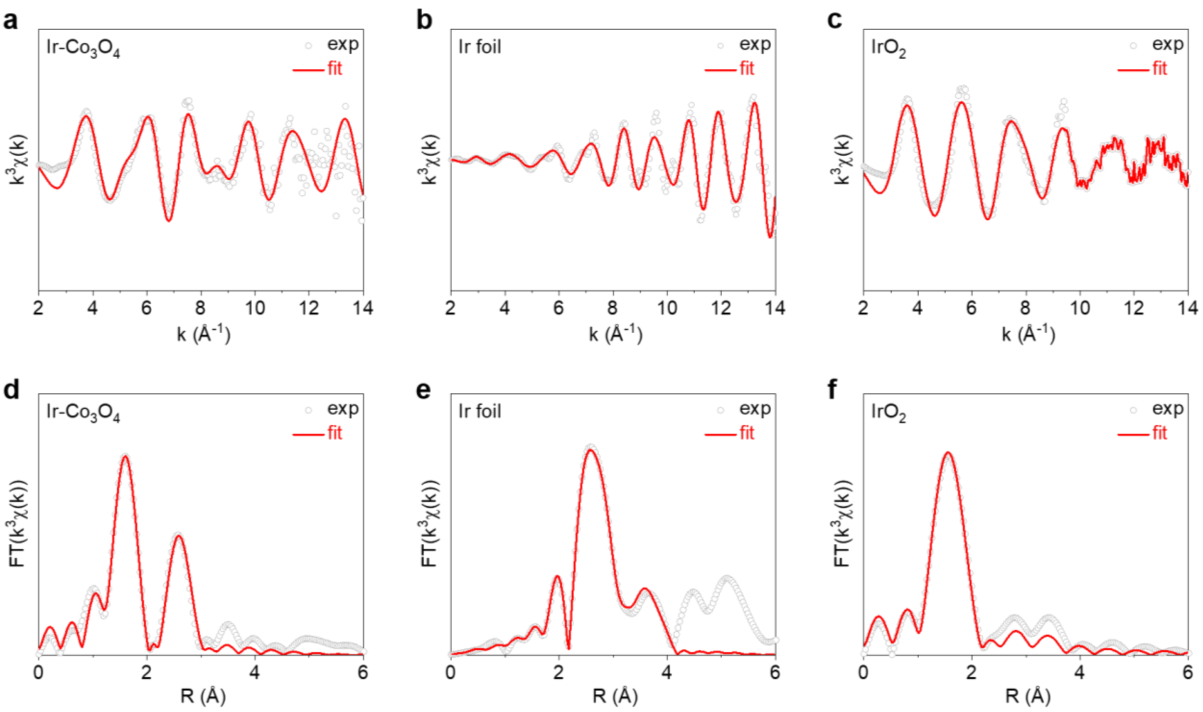

Supplementary Figure 9. (a-c) k^3-weighted k-space Ir L_3-edge experimental and fitting spectra of Ir-Co_3O_4 and references. (d-e) Corresponding k^3-weighted R-space Ir L_3-edge experimental and fitting spectra of Ir-Co_3O_4 and references.

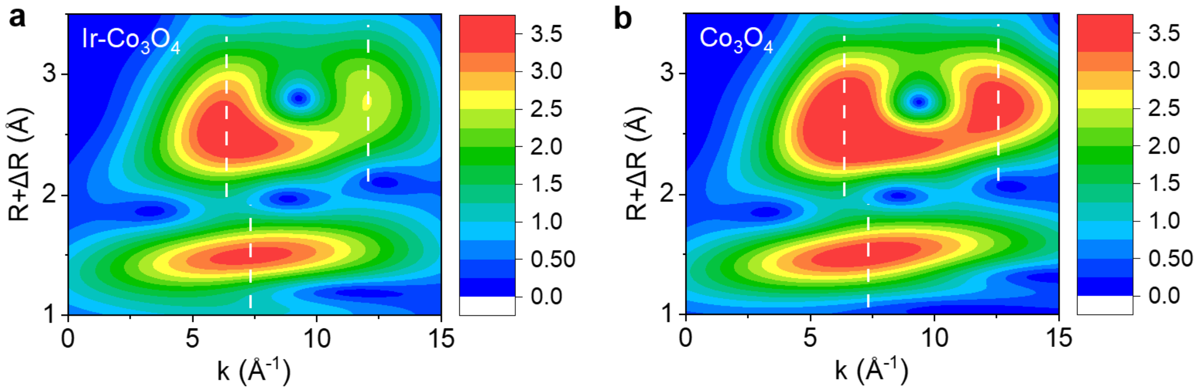

Supplementary Figure 10. WT-EXAFS analysis of Co *K*-edge on (a) Ir-Co_3O_4 and (b) Co_3O_4.

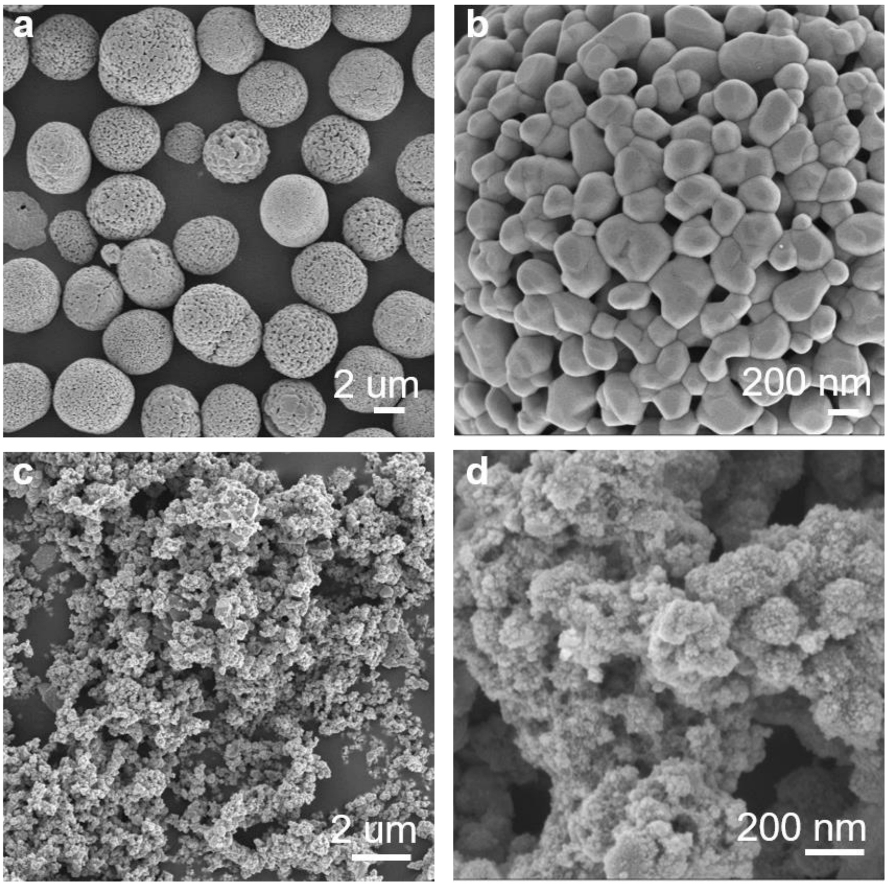

Supplementary Figure 11. SEM images of (a, b) commercial Co_3O_4 and (c, d) commercial IrO_2.

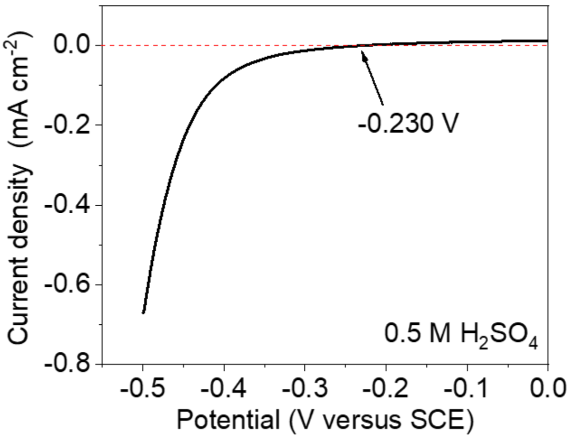

Supplementary Figure 12. Potential calibration of the calomel reference electrode at 25 ℃ in H_2-saturated 0.5 M H_2SO_4 solution.

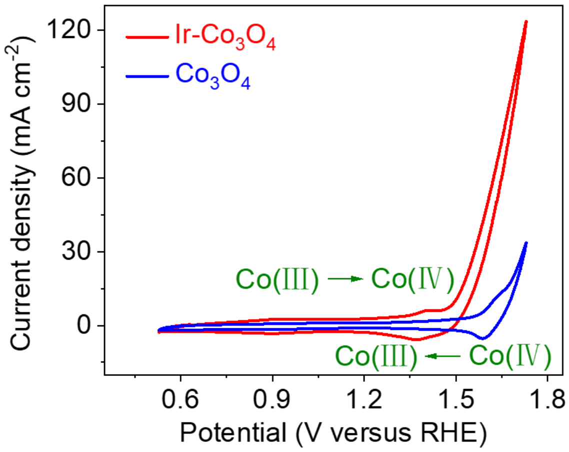

Supplementary Figure 13. CV curves within the Co redox potential range on Ir-Co_3O_4 and Co_3O_4 in 0.5 M H_2SO_4. Scan rates are 50 mV s^{-1}.

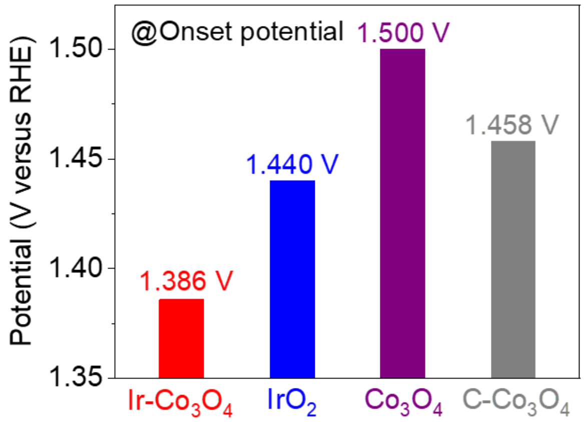

Supplementary Figure 14. The onset potentials for OER on Ir-Co_3O_4, IrO_2, Co_3O_4 and C-Co_3O_4.

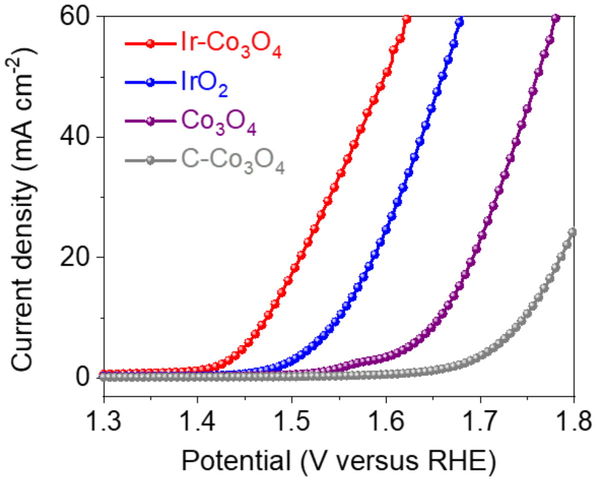

Supplementary Figure 15. Polarization curves without 95% iR compensation on Ir-Co_3O_4, IrO_2, Co_3O_4 and C-Co_3O_4 in 0.5 M H_2SO_4 at a scanning rate of 5 mV s^{-1}.

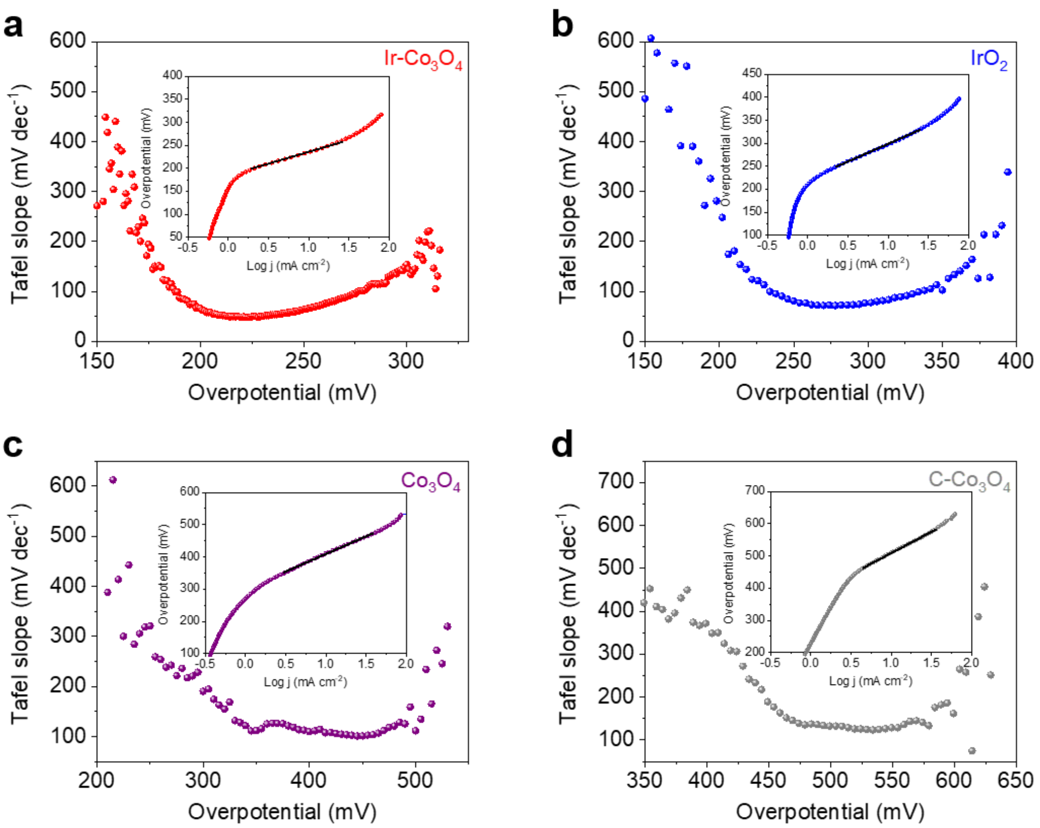

Supplementary Figure 16. The graphs that plot Tafel slope as a function of overpotential on (a) Ir-Co_3O_4, (b) IrO_2, (c) Co_3O_4 and (d) C-Co_3O_4. Insets are the Tafel plots of these catalysts.

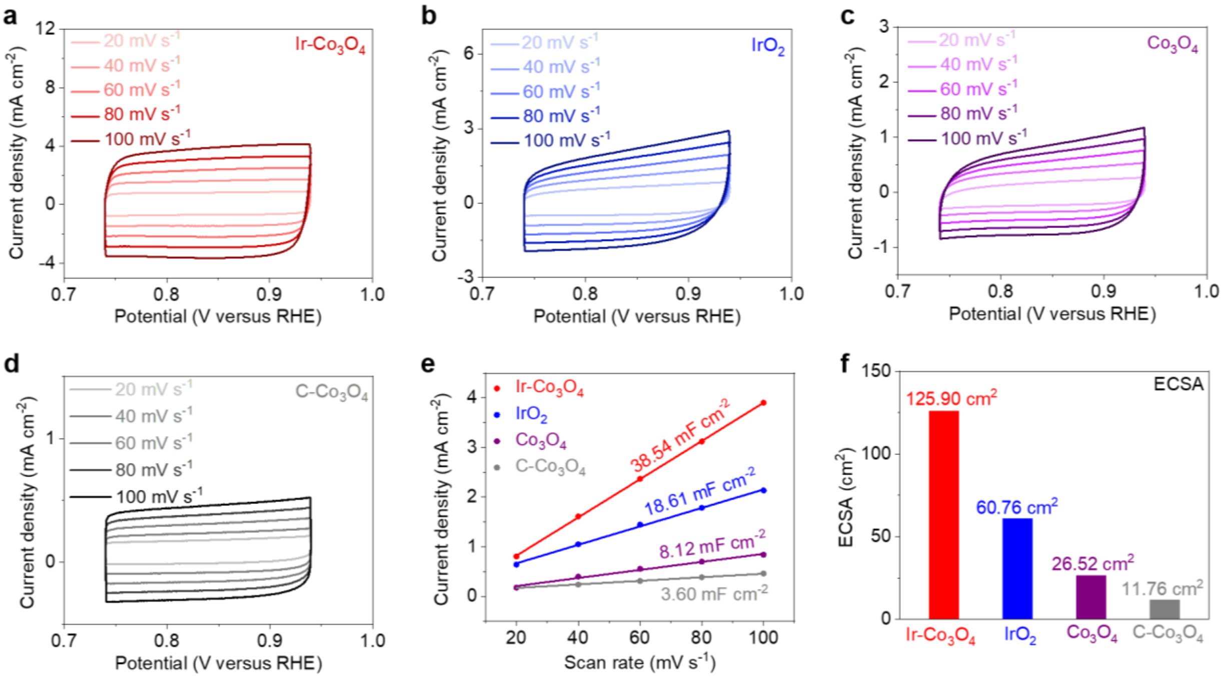

Supplementary Figure 17. Electrochemical cyclic voltammetry scans recorded for (a) Ir-Co_3O_4, (b) IrO_2, (c) Co_3O_4 and (d) C-Co_3O_4. Scan rates are 20, 40, 60, 80 and 100 mV s^{-1}. (e) Linear fitting of the capacitive currents versus cyclic voltammetry scans for these catalysts. (d) The calculated ECSA values for Ir-Co_3O_4, IrO_2, Co_3O_4 and C-Co_3O_4.

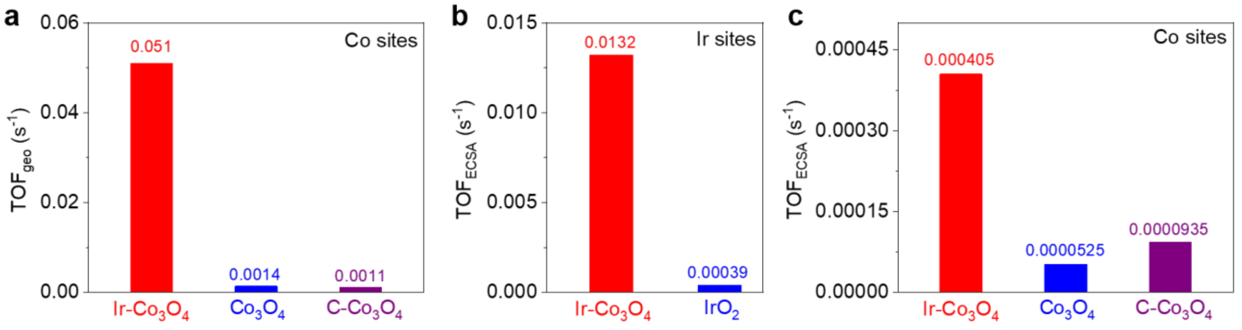

Supplementary Figure 18. (a) The TOF values that normalized to Co sites per geometric area of Ir-Co_3O_4, Co_3O_4 and C-Co_3O_4. The calculated TOF values that normalized to (a) Ir sites on Ir-Co_3O_4 and IrO_2 and (b) Co sites on Ir-Co_3O_4, Co_3O_4 and C-Co_3O_4 per ECSA.

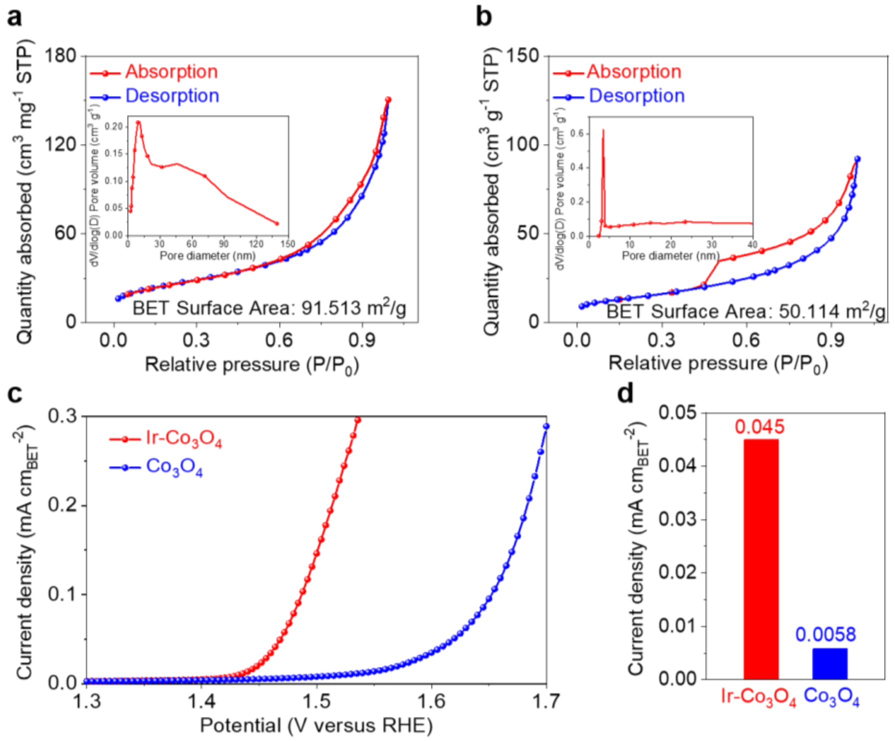

Supplementary Figure 19. N_2 absorption-desorption isotherms of (a) Ir-Co_3O_4 and (b) Co_3O_4. (c) The polarization curves normalized to BET surface areas and (d) corresponding normalized current densities of Ir-Co_3O_4 and Co_3O_4 at the overpotential of 236 mV.

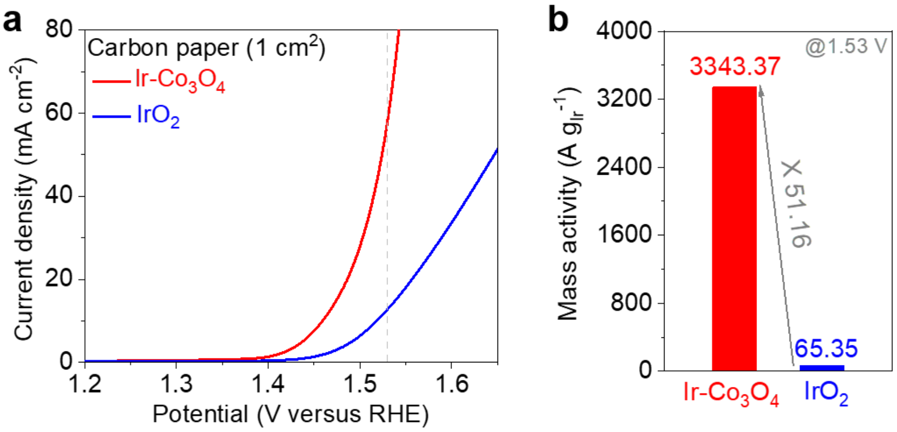

Supplementary Figure 20. (a) The OER polarization curves of Ir-Co_3O_4 and IrO_2 collected on the carbon paper electrode. (b) Corresponding mass activities of these catalysts at overpotential of 300 mV.

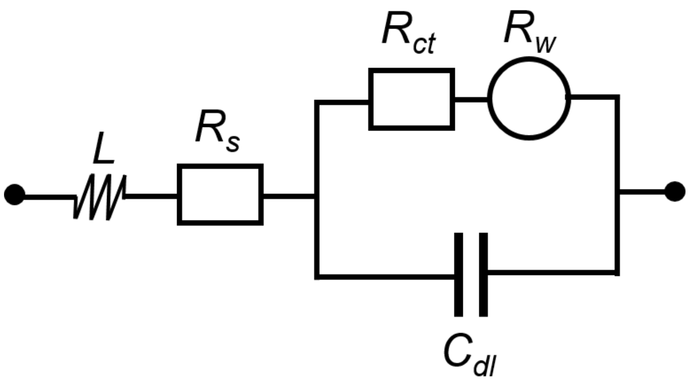

Supplementary Figure 21. The equivalent circuit applied in the EIS tests of these catalysts.

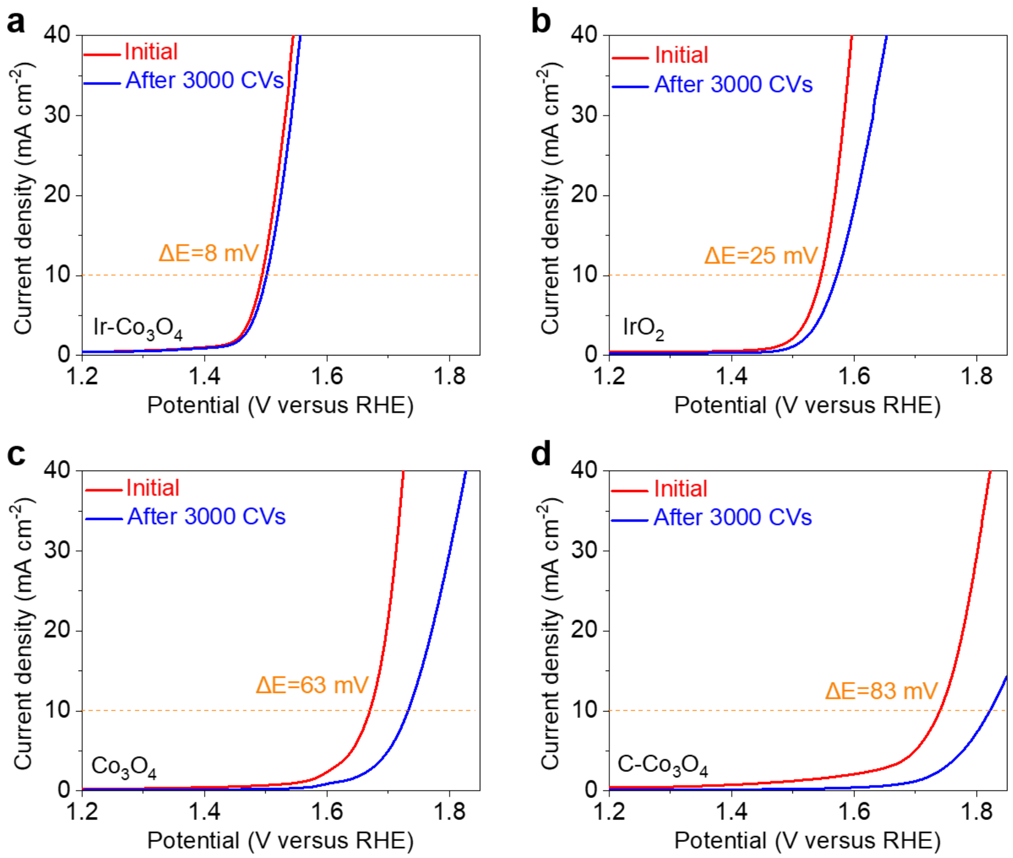

Supplementary Figure 22. Polarization curves of (a) Ir-Co_3O_4, (b) IrO_2, (c) Co_3O_4 and (d) C-Co_3O_4 before and after 3000 CV cycles in 0.5 M H_2SO_4 solution

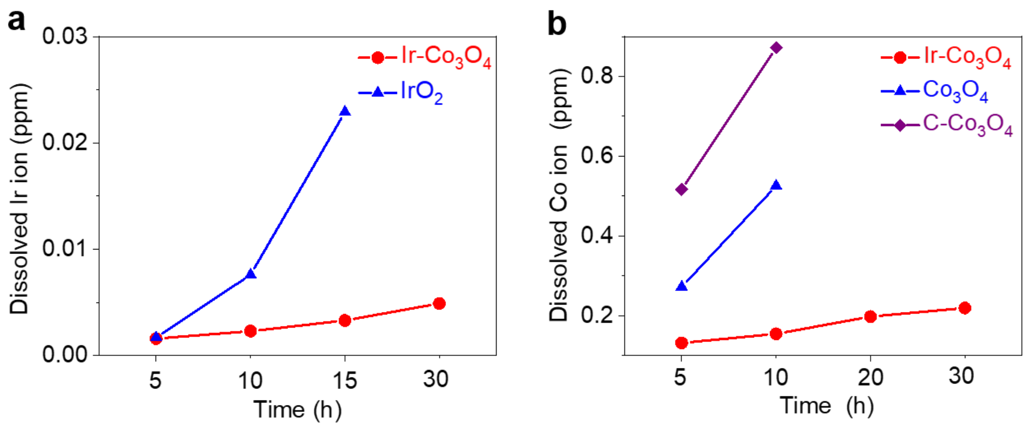

Supplementary Figure 23. (a) The dissolved Ir ion concentrations measured for Ir-Co_3O_4 and IrO_2, and (b) the dissolved Co ion concentrations measured for Ir-Co_3O_4, Co_3O_4 and C-Co_3O_4 in electrolyte during the chronopotentiometry at 10 mA cm^{-2} by ICP-OES.

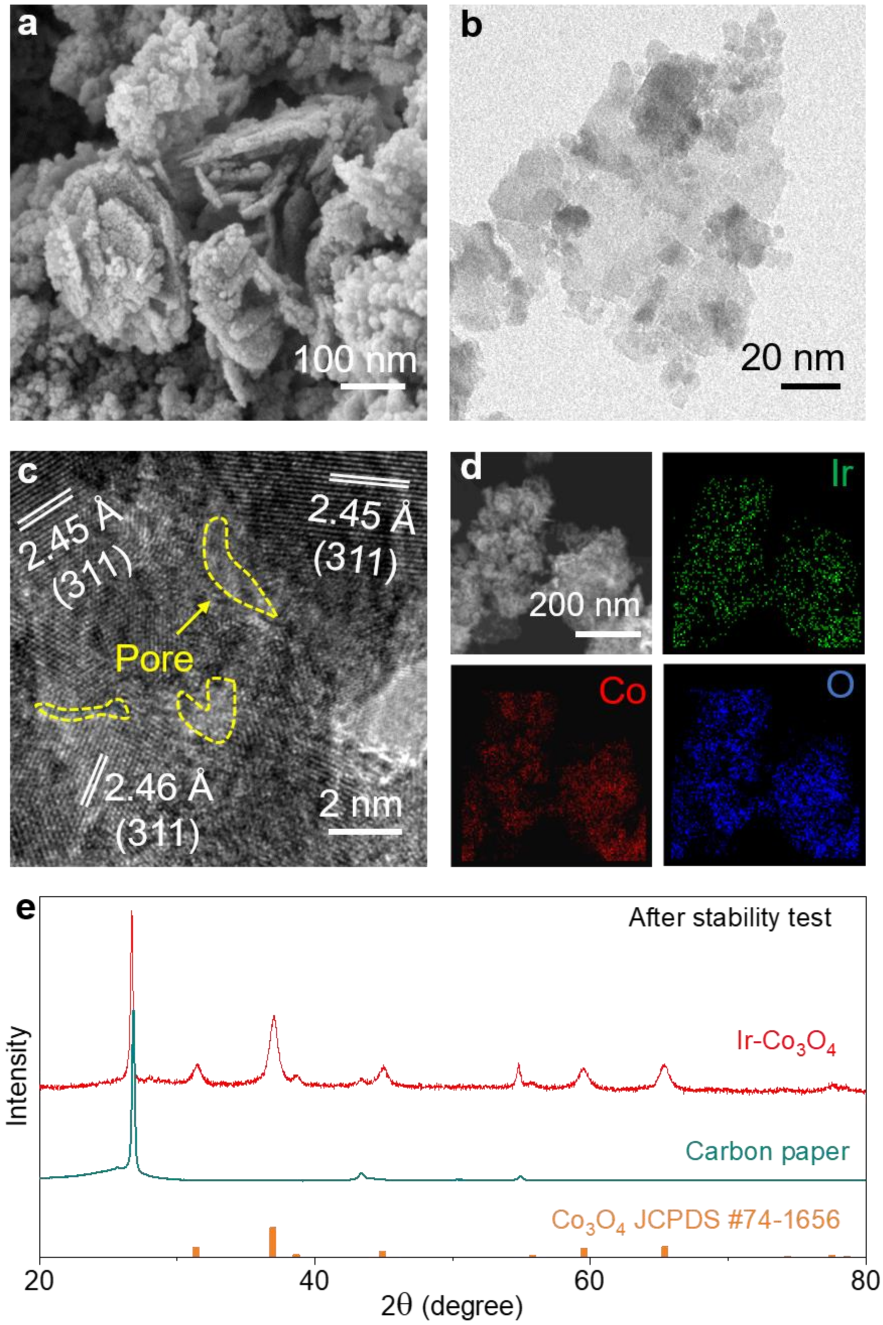

Supplementary Figure 24. Corresponding (a) SEM image, (b) TEM image, (c) HRTEM image, (d) elemental mappings and (e) XRD pattern of Ir-Co_3O_4 after stability test. Yellow circles represent the defective pores on Ir-Co_3O_4.

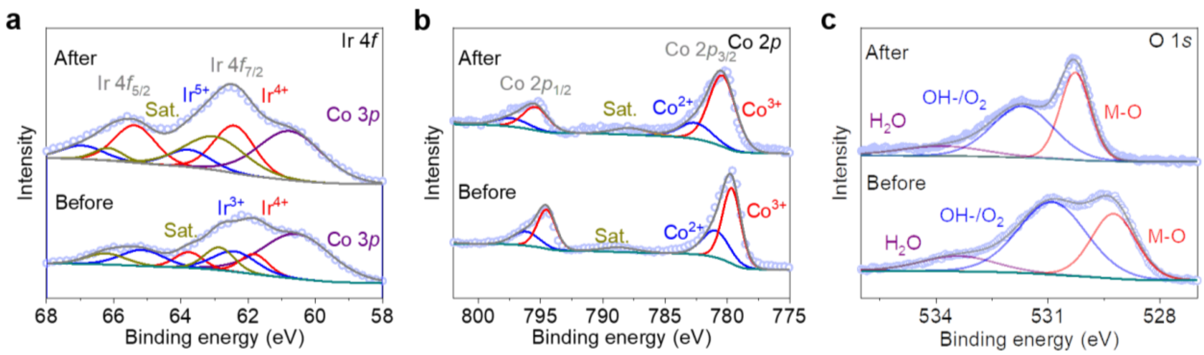

Supplementary Figure 25. Corresponding (a) Ir 4*f*, (b) Co 2*p*, (c) O1*s* XPS spectrum of Ir-Co_3O_4 before and after stability test.

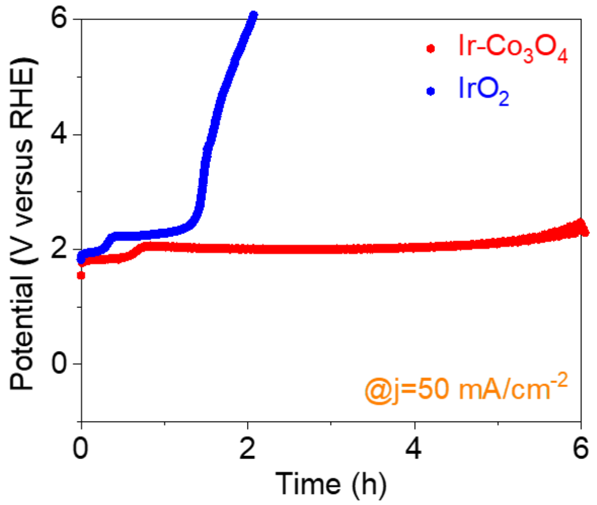

Supplementary Figure 26. Chronopotentiometric measurements of Ir-Co_3O_4 and IrO_2 at 50 mA cm^{-2}, carbon paper was used as the catalyst support.

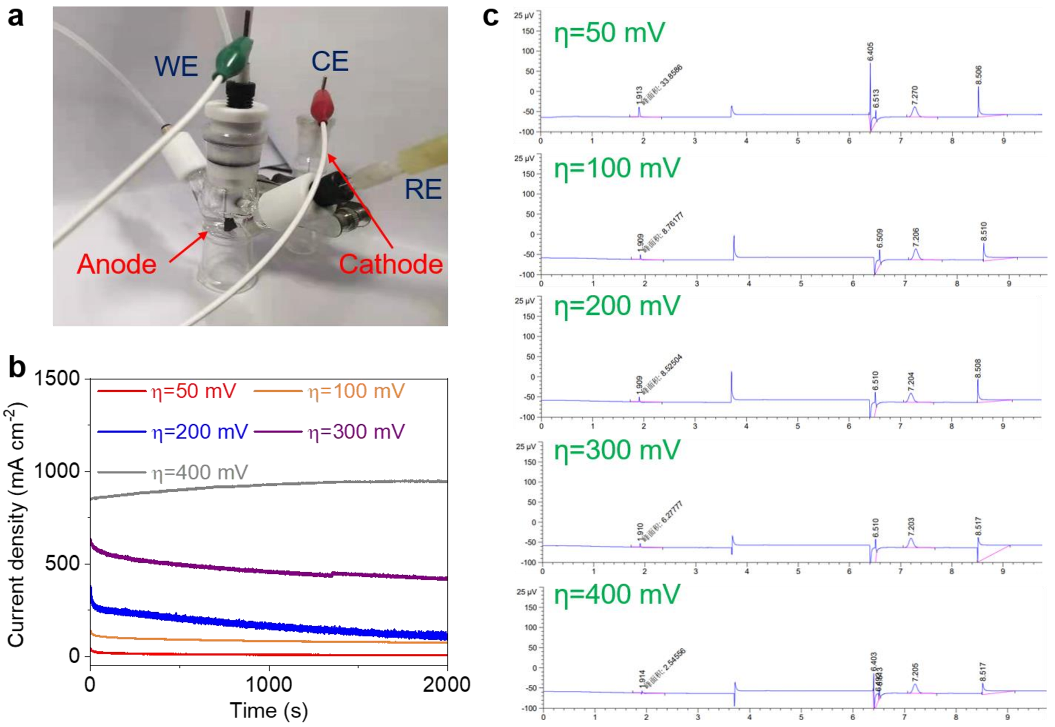

Supplementary Figure 27. (a) The setup used for conducting the OER on Ir-Co_3O_4 and was connected to GC to detect the amount of generated O_2. (b) Corresponding I-t curves of Ir-Co_3O_4 under various overpotentials for 2000s. (c) The GC signals of generated O_2 amount detected under various overpotentials on Ir-Co_3O_4.

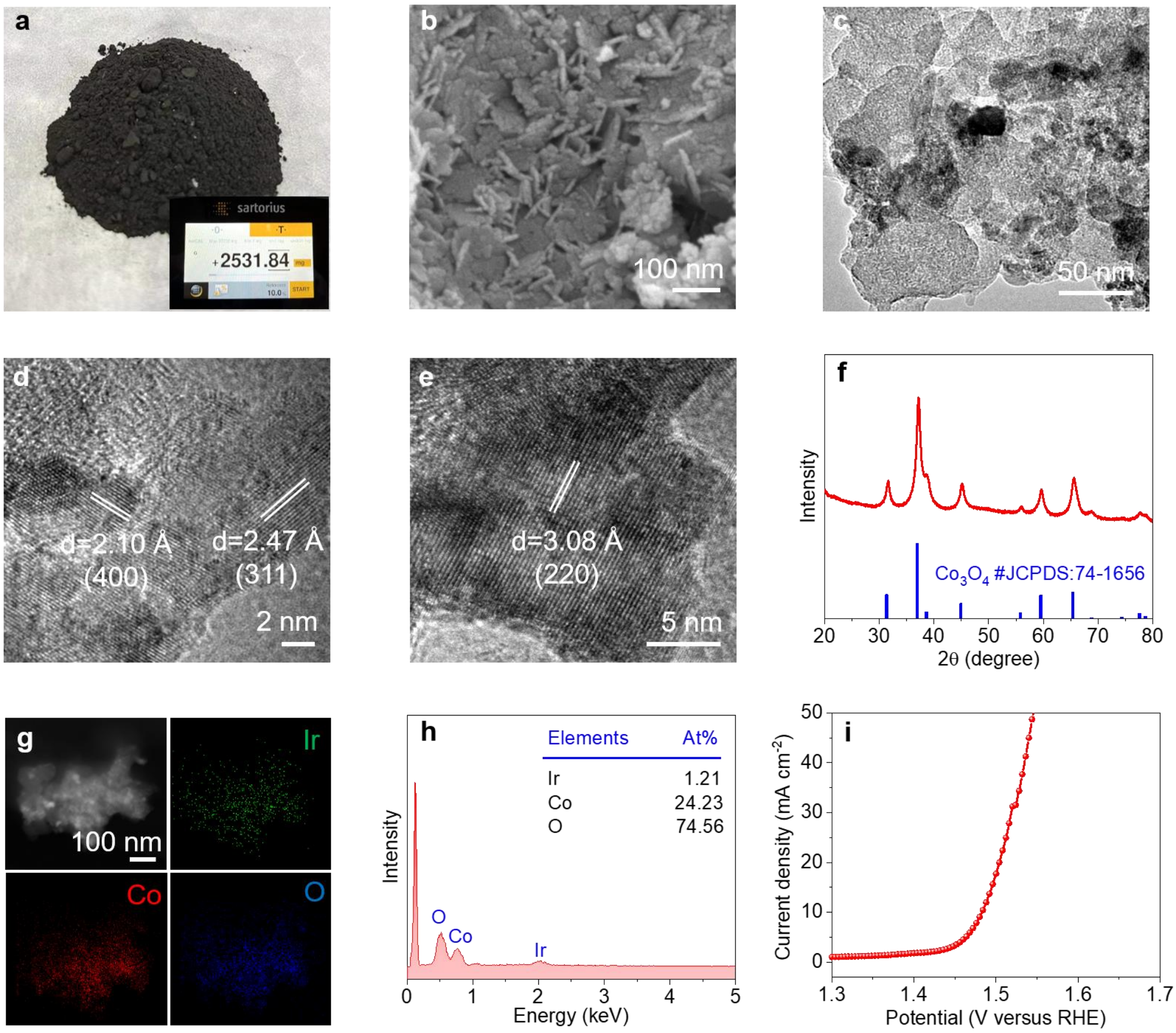

Supplementary Figure 28. (a) Digital photo, (b) SEM image, (c) TEM image, (d,e) HRTEM images, (f) XRD pattern, (g) elemental mappings, (h) SEM-EDS spextra and (i) polarization curves of the scale-up Ir-Co_3O_4.

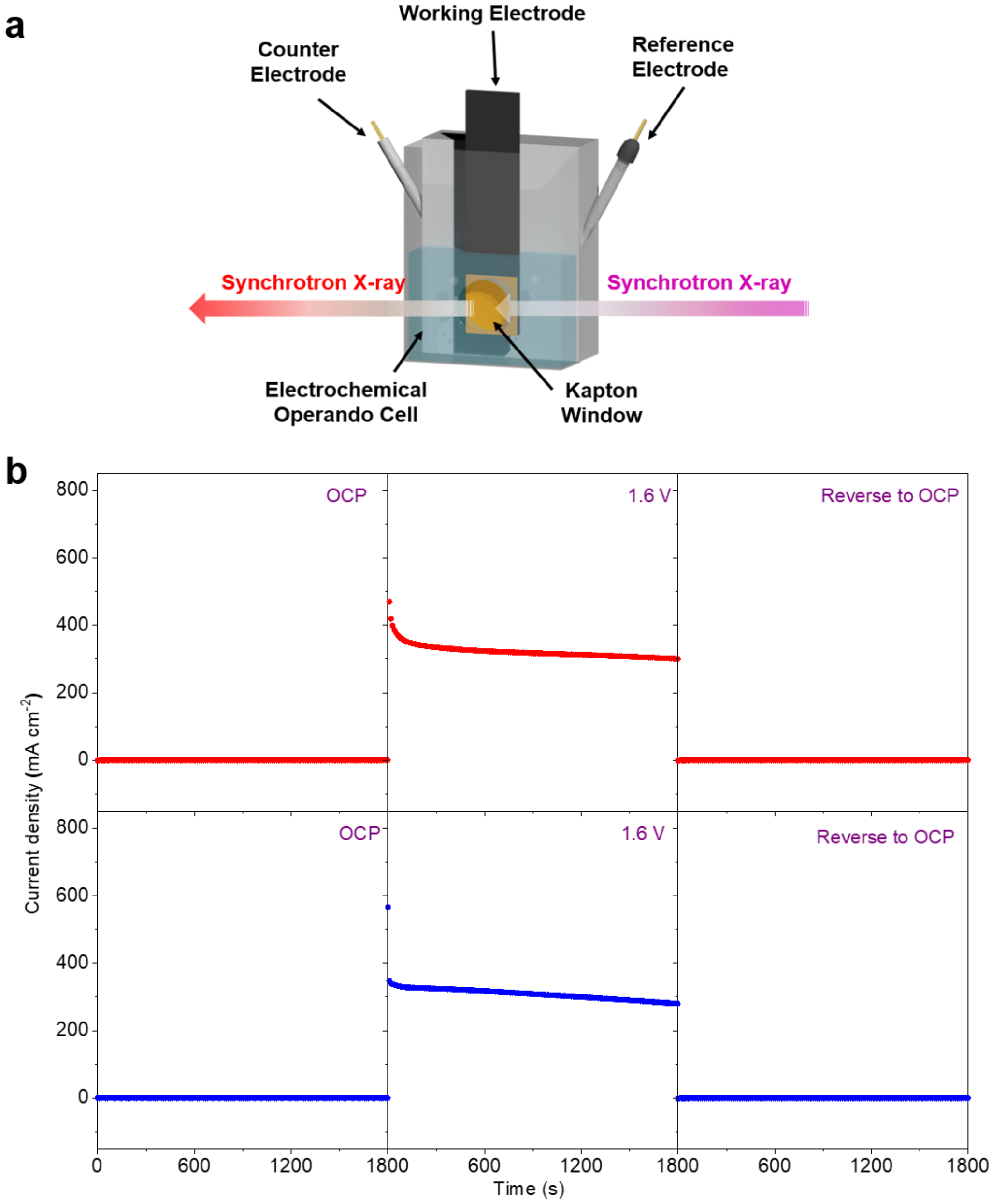

Supplementary Figure 29. (a) The schematic diagram of the custom-built electrochemical cell for operando XAS test. (b) The i-t curves were sequentially obtained under the applied potentials of OCP, 1.6 V and the reverse to OCP again, the Ir-Co_3O_4 was maintained under each potential for 1800 s. Red curves were conducted in the home-built electrochemical cell for collecting the operando XAS data and blue curves were conducted in the lab cell under the same condition for comparison, only slight difference can be distinguished between them.

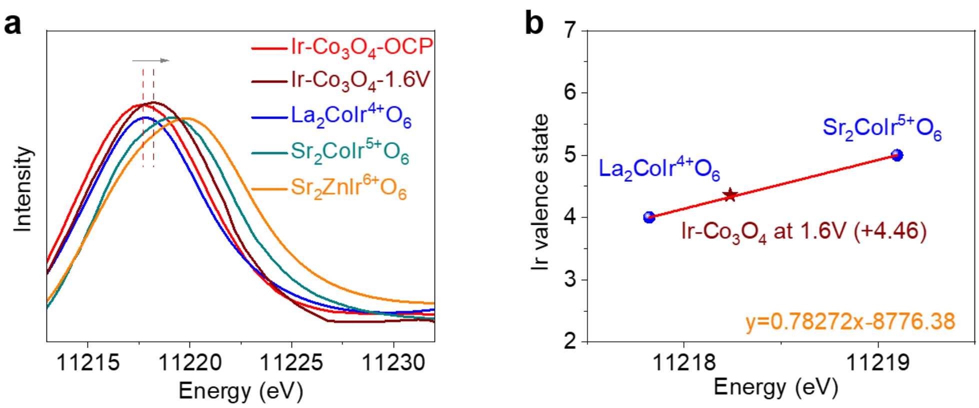

Supplementary Figure 30. (a) Operando Ir-L_3 XANES spectra of Ir-Co_3O_4 at OCP and 1.6 V. La_2CoIrO_6, Sr_2CoIrO_6 and Sr_2ZnIrO_6 represent the Ir^{4+}, Ir^{5+} and Ir^{6+} references, respectively. (b) Ir valence state-absorption energy standard curves determined by La_2CoIrO_6 and Sr_2CoIrO_6, and the calculated Ir valence state of Ir-Co_3O_4 at 1.6 V.

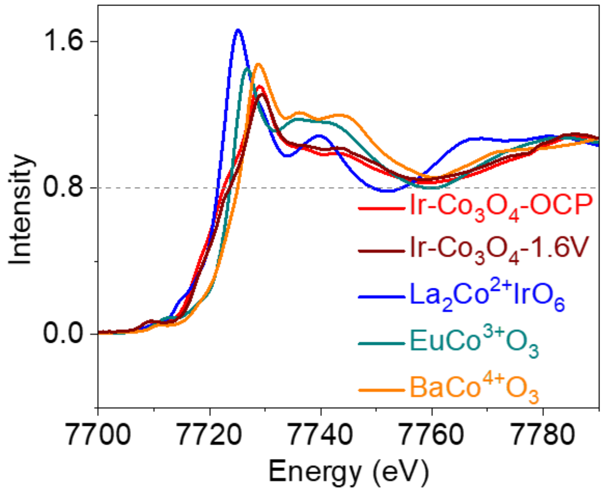

Supplementary Figure 31. Operando Co-*K* XANES spectra of Ir-Co_3O_4 at OCP and 1.6 V. La_2CoIrO_6, $EuCoO_3$ and $BaCoO_3$ represent the Co^{2+}, Co^{3+} and Co^{4+} references, respectively.

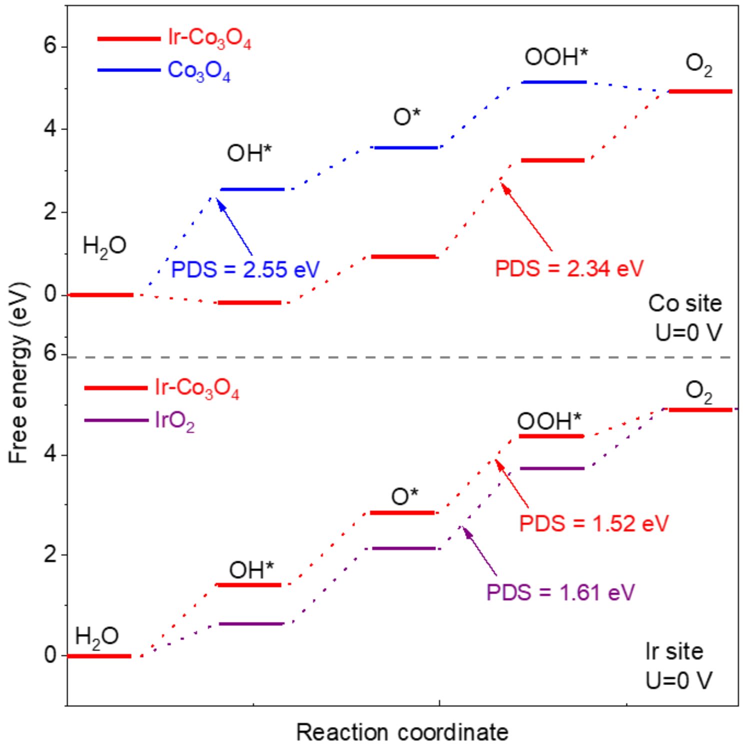

Supplementary Figure 32. The Gibbs free energy diagrams of the four-electron OER process on the Ir sites and Co sites of these catalysts under the applied overpotentials of 0 V vs. RHE, respectively.

Supplementary Table 1. Results of XRD refinements for Ir-Co_3O_4 and Co_3O_4.

Compounds	*a* (Å)	*b* (Å)	*c* (Å)	*V* (Å^3)
Ir-Co_3O_4	8.097	8.097	8.097	530.9
Co_3O_4	8.075	8.075	8.075	526.6

Supplementary Table 2. Fit goodness and R-factor of XRD refinements for Ir-Co_3O_4 and Co_3O_4.

Compounds	χ^2	R_F	R_B	R_P	R_{WP}
Ir-Co_3O_4	1.78	0.412%	0.561%	10.1%	6.41%
Co_3O_4	1.34	0.771%	0.919%	15.6%	9.05%

Supplementary Table 3. EXAFS fitting parameters of Ir-Co_3O_4, Ir foil and IrO_2.

Compounds	Path	N	S02	σ^2 / Å^2	R / Å	ΔE0	R-factor
Ir-Co_3O_4	Ir-O1	1.685	0.759	0.00779±0.009	1.769±0.0985	6.396	0.00207
	Ir-O2	3.370	1.519	0.00409±0.003	1.968±0.0222		
	Ir-O-Co	3.290	1.464	0.01115±0.005	2.910±0.0251		
Ir foil	Ir-Ir1	12	0.638	0.00269±0.003	2.707±0.0033	6.717	0.0065
	Ir-Ir2	6	0.638	0.00246±0.001	3.838±0.0725		
IrO_2	Ir-O	6	0.901	0.00650±0.003	2.012±0.0206	9.881	0.0186
	Ir-Ir	2	0.901	0.00801±0.009	3.151±0.0746		

Supplementary Table 4. Comparisons of the Tafel slopes and overpotentials at the current density of 10 mA cm^{-2} of reported Ir-based catalysts for OER in 0.5 M H_2SO_4.

Catalysts	Overpotential (mV)	Tafel slope (mV dec^{-1})	Ref.
Ir-$NiCo_2O_4$ NSs	240	60	1
IrO_2/GCN	276	57	2
IrO_x/9R-$BaIrO_3$	230	80	3
$Ru_3Ir_1O_X$	231	93.2	4
$Rh_{22}Ir_{78}$	292	101	5
Ir@Fe/NCNT	250	58.2	6
DO-$IrTe_2$	298	49.4	7
$Au_{0.5}Ir_{0.5}$	257	77.6	8
Ir_6Ag_9 NTs	285	61.1	9
Mesoporous Ir NSs	240	49	10
P-IrO_X@DG	290	67.5	11
IrO_2	298	82	**This work**
Ir-Co_3O_4	236	52.6	

Supplementary Table 5. Fitting parameters of EIS for Ir-Co_3O_4, IrO_2, Co_3O_4 and C-Co_3O_4.

Samples	R_s (Ω)	R_{ct} (Ω)	C_{dl} (F s^{n-1})	s (Ω s$^{-1/2}$)
Ir-Co_3O_4	4.41	2.37	0.0001	0.001
IrO_2	4.24	4.18	0.015	0.0015
Co_3O_4	4.80	20.55	0.005	0.001
C-Co_3O_4	4.31	115.5	0.001	0.001

Supplementary References

1. Yin, J. et al. Iridium single atoms coupling with oxygen vacancies boosts oxygen evolution reaction in acid media. *J. Am. Chem. Soc.* **142**, 18378-18386 (2020).
2. Chen, J. et al. Low-coordinate iridium oxide confined on graphitic carbon nitride for highly efficient oxygen evolution. *Angew. Chem. Int. Ed.* **58**, 12540-12544 (2019).
3. Li, N. et al. Identification of the active-layer structures for acidic oxygen evolution from 9R-$BaIrO_3$ electrocatalyst with enhanced iridium mass activity. *J. Am. Chem. Soc.* **143**, 18001-18009 (2021).
4. He, J., Zhou, X., Xu, P. & Sun, J. Regulating electron redistribution of intermetallic iridium oxide by incorporating Ru for efficient acidic water oxidation. *Adv. Energy Mater.* **11**, 2102883 (2021)
5. Guo, H. et al. Rational design of rhodium-iridium alloy nanoparticles as highly active catalysts for acidic oxygen evolution. *ACS Nano* **13**, 13225-13234 (2019).
6. Luo, F. et al. Robust and stable acidic overall water splitting on Ir single atoms. *Nano Lett.* **20**, 2120-2128 (2020).
7. Pi, Y. et al. Selective surface reconstruction of a defective iridium-based catalyst for high-efficiency water splitting. *Adv. Funct. Mater.* **30**, 2004375 (2020).
8. Hu, H. et al. Electronically delocalized Ir enables efficient and stable acidic water splitting. *J. Mater. Chem. A* **8**, 20168 (2020).
9. Zhu, M., Shao, Q., Qian, Y. & Huang, X. Superior overall water splitting electrocatalysis in acidic conditions enabled by bimetallic Ir-Ag nanotubes. *Nano Energy* **56**, 330-337 (2019).
10. Jiang, B. et al. Mesoporous metallic iridium nanosheets. *J. Am. Chem. Soc.* **140**, 12434-12441 (2018).
11. Zhuang, L. et al. Porous structure engineering of iridium oxide nanoclusters on atomic scale for efficient pH-universal overall water splitting. *Small* **17**, 2100121 (2021).
